# Daily Step Count and Depression in Adults: A Systematic Review and Meta-Analysis

**DOI:** 10.1001/jamanetworkopen.2024.51208

**Published:** 2024-12-16

**Authors:** Bruno Bizzozero-Peroni, Valentina Díaz-Goñi, Estela Jiménez-López, Eva Rodríguez-Gutiérrez, Irene Sequí-Domínguez, Sergio Núñez de Arenas-Arroyo, José Francisco López-Gil, Vicente Martínez-Vizcaíno, Arthur Eumann Mesas

**Affiliations:** 1 Health and Social Research Center, Universidad de Castilla-La Mancha, Cuenca, Spain; 2 Higher Institute of Physical Education, Universidad de la República, Rivera, Uruguay; 3 Centro de Investigación Biomédica en Red de Salud Mental, Instituto de Salud Carlos III, Madrid, Spain; 4 Research Network on Chronicity, Primary Care and Health Promotion (RICAPPS), Cuenca, Spain; 5 One Health Research Group, Universidad de Las Américas, Quito, Ecuador; 6 Faculty of Health Sciences, Universidad Autónoma de Chile, Talca, Chile

## Abstract

**Importance:**

Recent evidence syntheses have supported the protective role of daily steps in decreasing the risk of cardiovascular disease and all-cause mortality. However, step count–based recommendations should cover additional health outcomes.

**Objective:**

To synthesize the associations between objectively measured daily step counts and depression in the general adult population.

**Data Sources:**

In this systematic review and meta-analysis, a systematic search of the PubMed, PsycINFO, Scopus, SPORTDiscus, and Web of Science databases was conducted from inception until May 18, 2024, to identify observational studies using search terms related to physical activity, measures of daily steps, and depression, among others. Supplementary search methods were also applied.

**Study Selection:**

All identified studies were uploaded to an online review system and were considered without restrictions on publication date or language. Included studies had objectively measured daily step counts and depression data.

**Data Extraction and Synthesis:**

This systematic review and meta-analysis followed the Preferred Reporting Items for Systematic Reviews and Meta-analyses and Meta-analysis of Observational Studies in Epidemiology reporting guidelines. Two independent reviewers extracted the published data.

**Main Outcomes and Measures:**

Pooled effect sizes (correlation coefficient, standardized mean difference [SMD], and risk ratio [RR]) with 95% CIs were estimated using the Sidik-Jonkman random-effects method.

**Results:**

Thirty-three studies (27 cross-sectional and 6 longitudinal [3 panel and 3 prospective cohort]) involving 96 173 adults aged 18 years or older (range of mean [SD] ages: 18.6 [0.6] to 91.2 [1.6] years) were included. Daily steps were inversely correlated with depressive symptoms in both cross-sectional and panel studies. Compared with fewer than 5000 steps/d, pooled SMDs from cross-sectional studies revealed that 10 000 or more steps/d (SMD, −0.26; 95% CI, −0.38 to −0.14), 7500 to 9999 steps/d (SMD, −0.27; 95% CI, −0.43 to −0.11), and 5000 to 7499 steps/d (SMD, −0.17; 95% CI, −0.30 to −0.04) were significantly associated with fewer depressive symptoms. Pooled estimates from prospective cohort studies indicated that participants with 7000 or more steps/d had reduced risk of depression compared with their counterparts with fewer than 7000 steps/d (RR, 0.69; 95% CI, 0.62-0.77). An increase of 1000 steps/d was associated with a lower risk of depression (RR, 0.91; 95% CI, 0.87-0.94).

**Conclusions and Relevance:**

In this systematic review and meta-analysis of 33 observational studies involving 96 173 adults, higher daily step counts were associated with fewer depressive symptoms in cross-sectional and longitudinal studies in the general adult population. Further prospective cohort studies are needed to clarify the potential protective role of daily steps in mitigating the risk of depression during adulthood.

## Key Points

**Question:**

Are objectively measured daily steps associated with depression in adulthood?

**Findings:**

In this systematic review and meta-analysis of 33 observational studies involving 96 173 adults, higher daily step counts were associated with fewer depressive symptoms in the general adult population. Compared with fewer than 5000 steps/d, achieving 5000 or more was associated with reduced depressive symptoms in cross-sectional studies, whereas a daily step count of 7000 or higher was associated with lower risk of depression in prospective studies.

**Meaning:**

These findings suggest that an inclusive, comprehensive public health approach could contribute to preventing depression in adults.

## Teaser

This systematic review and meta-analysis of observational studies examines associations between objectively measured daily step counts and depression in the general adult population.

## Introduction

Depressive disorders are among the major causes of disability burden related to mental illness from early adulthood through older age,^1^ affecting over 330 million individuals worldwide.^2^ In addition, even when depressive symptoms are below the clinical threshold, they have a significant association with quality of life and a considerable likelihood of progressing to clinical depression.^3^ The etiology of depression involves a complex interplay of factors, ranging from biological to lifestyle related, which poses a challenge for primary prevention strategies.^4^

A 2020 meta-review summarized the critical role of modifiable health behaviors, such as physical activity (PA), in public health strategies focused on depression prevention.^5^ The results of meta-analytic studies have consistently indicated that higher levels of PA provide protection against the emergence of depression.^6,7^ Light-intensity activities, such as walking, may constitute an adequate form of PA for reducing the risk of depression.^8^ Although quantifying PA data from the general population is challenging,^7^ a genome-wide association study highlighted the importance of objectively assessing PA in epidemiologic studies focusing on mental health to elucidate the association between PA and depression.^9^

The number of daily steps is a simple and intuitive objective measure of PA.^10^ Currently, daily step monitoring is increasingly feasible for the general population as wearable devices have become more popular.^11,12^ Recent meta-analyses have provided evidence that higher step counts are associated with reduced risk of cardiovascular disease^13^ and all-cause mortality.^14^ However, the 2018 US Department of Health and Human Services^15^ and 2020 World Health Organization^16^ expert committees on PA guidelines recognized the need for further research to encompass additional health outcomes. To our knowledge, the association between the number of daily steps measured with wearable trackers and depression has not been previously examined through a meta-analytic approach. Setting PA goals based on step counts is straightforward to understand and integrate into daily routines and may be valuable to consider in depression prevention guidelines.^5^ Therefore, this study synthesized the available evidence from observational studies on the associations between objectively measured daily steps and depression in the general adult population.

## Methods

This systematic review and meta-analysis was performed in accordance with the 2020 Preferred Reporting Items for Systematic Reviews and Meta-Analyses (PRISMA)^17^ and the Meta-analysis of Observational Studies in Epidemiology (MOOSE)^18^ reporting guidelines. The study protocol was registered in PROSPERO. Two researchers (B.B.-P. and V.D.-G.) independently performed the literature search, screening, study selection, data extraction, and methodological quality assessment. Disagreements were resolved by consulting a third researcher (A.E.M.). A condensed section of the methods is presented herein, and the full methods are available in the eAppendix in Supplement 1.

### Data Sources and Search Strategy

Systematic searches were conducted in the following electronic databases from inception to July 14, 2023: PsycINFO, PubMed, Scopus, SPORTDiscus, and Web of Science. The search was later updated to include the period from July 1, 2023, to May 18, 2024. Supplementary search methods were performed on specific information sources, such as online resources (Google Scholar) or citation searching (references of included studies and relevant systematic reviews). The full approach to search strategies, including search terms, is detailed in eTable 1 in Supplement 1.

### Eligibility Criteria

To be included, studies retrieved from the peer-reviewed literature must have reported the following: (1) the general adult population (≥18 years of age) as participants, (2) daily step count data obtained through objective measuring devices (accelerometers, pedometers, or smartphones) and the exposure presented as either a continuous variable or a categorical daily step count data (high vs low numbers of daily steps), (3) depression as a diagnosis or depressive symptoms as the outcome, and (4) observational study design (cross-sectional, case-control, or longitudinal) published in an academic journal. No language, publication date, or other restrictions were applied.

### Study Selection

All studies identified were uploaded to the Rayyan review system online^19^ and underwent deduplication. Next, a 2-step process was applied. First, based on title and abstract, studies that did not address the association between daily steps and depression in the general adult population were excluded. Second, the remaining studies were analyzed by reading the full text to determine whether they met the eligibility criteria.

### Data Extraction and Quality Assessment

The data extracted from the included studies are detailed in the eAppendix in Supplement 1. The methodological quality of both cross-sectional and longitudinal studies was evaluated using the National Heart, Lung, and Blood Institute Quality Assessment Tool for Observational Cohort and Cross-Sectional Studies.^20^ The details of the quality assessment tool for grading observational studies are summarized in the eAppendix in Supplement 1.

### Exposure Harmonization

To facilitate interpretation and comparison between studies, we performed separate analyses depending on whether the exposure was continuous (higher number of steps per day) or categorical (high vs low counts of steps per day). The daily step categories were harmonized according to the classification system of Tudor-Locke et al^21^ (eAppendix in Supplement 1).

### Statistical Analysis

#### Effect Sizes

The pooled correlation coefficients (*r*) with their 95% CIs were estimated using Fisher *z* transformation. When studies compared daily step categories (high vs low counts [reference]), we calculated the standardized mean differences (SMDs) and their 95% CIs using the Cohen *d* index.^22^ Odds ratios (ORs) and risk ratios (RRs) were computed only for studies reporting depression as a categorical outcome. The ORs, *r* values, and SMDs were determined according to the data or estimator used in each study by applying the appropriate formula.^23,24,25,26^ All effect sizes and main results of the included studies are listed in eTable 2 in Supplement 1.

#### Data Synthesis

A meta-analysis for each study design (cross-sectional or longitudinal [panel study or prospective cohort]) and exposure harmonization (continuous or categorical) was conducted using a random-effects model with the Sidik-Jonkman method. Heterogeneity was assessed using the *I*^2^ metric, categorized as not important (0%-40%), moderate (30%-60%), substantial (50%-90%), or considerable (75%-100%).^26^ The corresponding *P* values were also considered. In addition, 95% prediction intervals were reported to reflect the amount of variation the results of a future study might have.^27^

Subgroup analyses were performed according to adult age group (18-35 years, 36-64 years, or ≥65 years), sex, step counter device, position and acceleration axis, and methodological quality of the studies. Univariate random-effects meta-regression models were used to examine whether participant characteristics influenced effect size estimates and between-study heterogeneity, including mean age, sex, and mean body mass index (BMI).

Sensitivity analyses were conducted using the leave-one-out method^26^ to assess the robustness of the summary estimates. Additional analyses were performed by excluding studies with specific data on the proposed association during the COVID-19 lockdown.^28,29^ Furthermore, we estimated the pooled ORs between high (≥7500) vs low (<7500) counts of daily steps and depression as a categorical outcome.^30,31,32,33^ Publication bias was assessed using the Egger regression asymmetry test and evaluating funnel plots through visual inspection.^34^

Statistical significance was set at 2-sided *P* < .05. All analyses were conducted via R, version 4.3.2 (R Project for Statistical Computing) with the meta^35^ and metafor^36^ packages. Further methodological considerations regarding data collection and analysis are detailed in the eAppendix in Supplement 1.

## Results

### Study Selection

**Figure 1.**
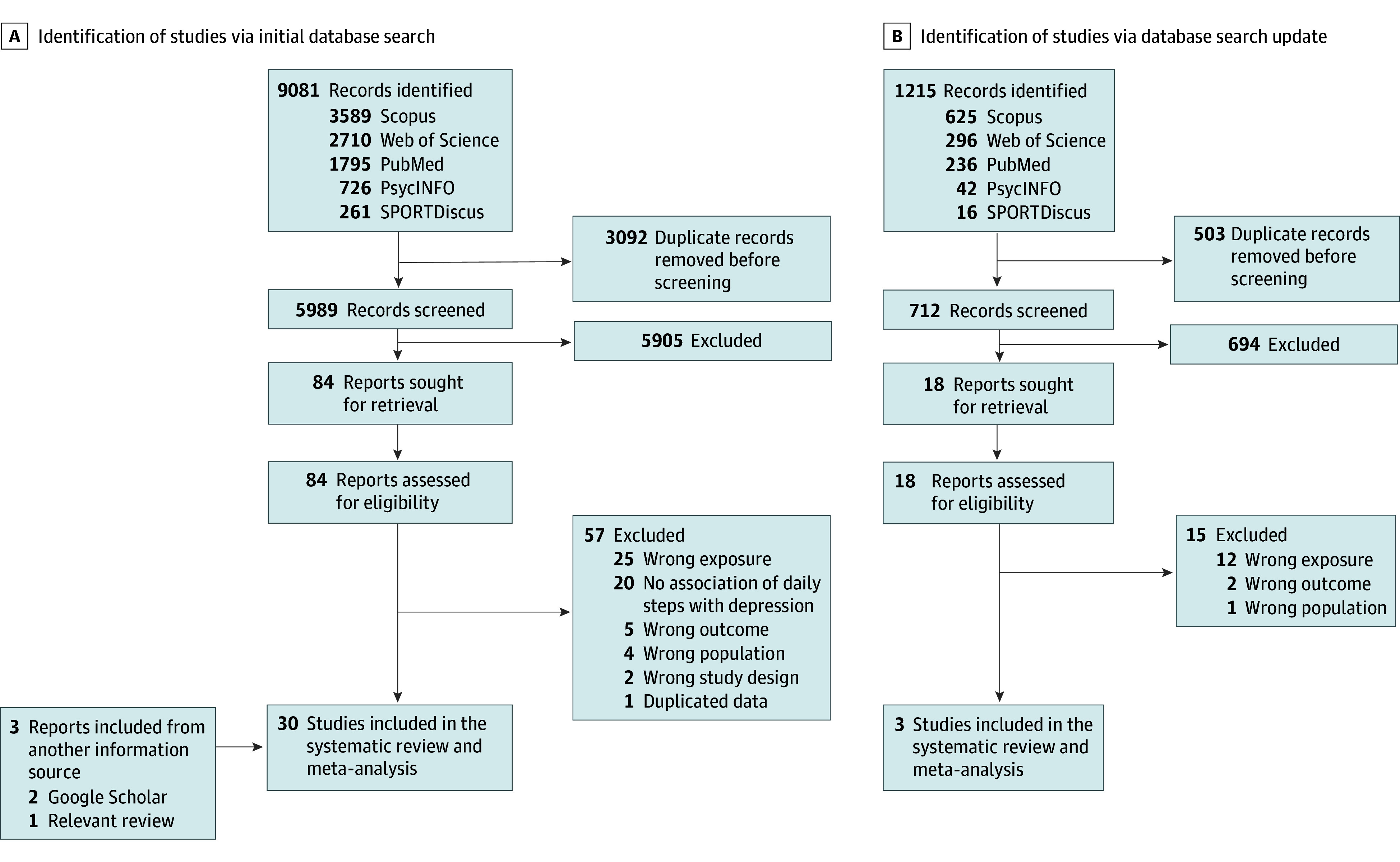
Flow Diagram of Study Selection

A total of 10 296 studies were considered for title and abstract review after removing duplicates. Of these, 102 were fully assessed for eligibility and 72 were finally excluded (eTable 3 in Supplement 1). A total of 33 studies were included in the systematic review, consisting of 27 cross-sectional studies^28,29,30,31,32,33,37,38,39,40,41,42,43,44,45,46,47,48,49,50,51,52,53,54,55,56,57^ and 6 longitudinal studies^58,59,60,61,62,63^ (3 panel studies^58,59,61^ and 3 prospective cohort studies^60,62,63^) (Figure 1). Three studies were included based on supplementary search methods.^53,54,58^

### Study Characteristics

eTables 4 and 5 in Supplement 1 summarize the main characteristics of the included studies. The studies were published between 2004^59^ and 2023.^52,56,57,63^ Among the longitudinal studies, the mean interval between the first and last waves ranged from 2^58,59^ to 5^61^ years for the panel studies, and the mean (SD) follow-up ranged from 1.8 (0.1)^60^ to 7.4 (1.1)^63^ years for the prospective cohort studies.

### Population

The studies included a total of 96 173 adults (54.5% female; 45.5% male) in 13 different countries from Asia,^38,43,44,45,46,48,52,59,60,61^ Europe,^28,29,33,37,50,55,56,57,58,63^ North America,^30,32,39,40,41,42,47,49,53,54,62^ Oceania,^31,51^ and South America.^42,47^ The mean (SD) age ranged from 18.6 (0.6)^52^ to 91.2 (1.6) years,^56^ including adults aged 18 to 35 years,^29,31,42,44,45,47,52^ 36 to 64 years,^28,30,37,39,46,50,63^ and 65 years or older.^32,33,38,40,41,43,48,49,51,56,57,58,59,60,61,62^ The cohort studies excluded participants who reported depression at the baseline assessment^62,63^ or performed analyses that excluded participants with mild to severe depressive symptoms.^60^

### Exposure and Outcome

The mean (SD) number of daily steps ranged from 2931 (2448)^56^ to 10 378 (1120).^28^ The daily step count was estimated using accelerometer^28,29,30,32,37,38,39,40,42,43,44,45,46,48,49,50,52,53,54,55,56,57,60,61,62,63^ or pedometer^31,33,41,47,51,58,59,62^ devices. The measurement period used was 7 days in most studies, ranging from 3^40,41^ to 365^38^ days. Exposure harmonization was reported as the higher number of daily steps^28,29,33,38,39,40,42,43,44,45,50,52,53,54,55,56,57,58,59,60,62,63^ and as the comparison between daily step categories.^30,31,32,33,37,41,46,47,48,49,50,51,60,61,62^ Depression was reported as a diagnosis (categorical variable)^31,62,63^ or as self-reported symptoms (number of depressive symptoms as a continuous variable^28,29,33,37,38,39,40,41,42,43,44,45,47,48,49,50,51,52,56,57,58,59,60,61^ or mild to severe depressive symptoms as a categorical variable^30,32,33,46^).

### Quality Assessment

Among the cross-sectional studies, 6 (22.2%) were rated as good quality,^30,31,32,37,47,51^ 19 (70.4%) as fair,^28,29,33,39,40,41,42,43,44,45,46,48,49,50,52,53,55,56,57^ and 2 (7.4%)^38,54^ as poor. Among the longitudinal studies, 2 (33.3%) were rated as good quality^60,62^ and 4 (66.7%) as fair^58,59,61,63^ (eTable 6 in Supplement 1).

### Meta-Analysis

Twenty-nine studies with cross-sectional data^28,29,30,31,32,33,37,38,39,40,41,42,43,44,45,47,48,49,50,51,52,53,54,55,56,57,58,60,61^ from 14 260 individuals (mean [SD] age range, 18.6 [0.6] to 91.2 [1.6] years) were included in the meta-analyses. Six longitudinal studies^58,59,60,61,62,63^ from 78 655 individuals (mean [SD] age range, 55.2 [19.4] to 74.5 [6.1] years) were included in the meta-analyses.

### Daily Steps as a Continuous Variable

**Figure 2.**
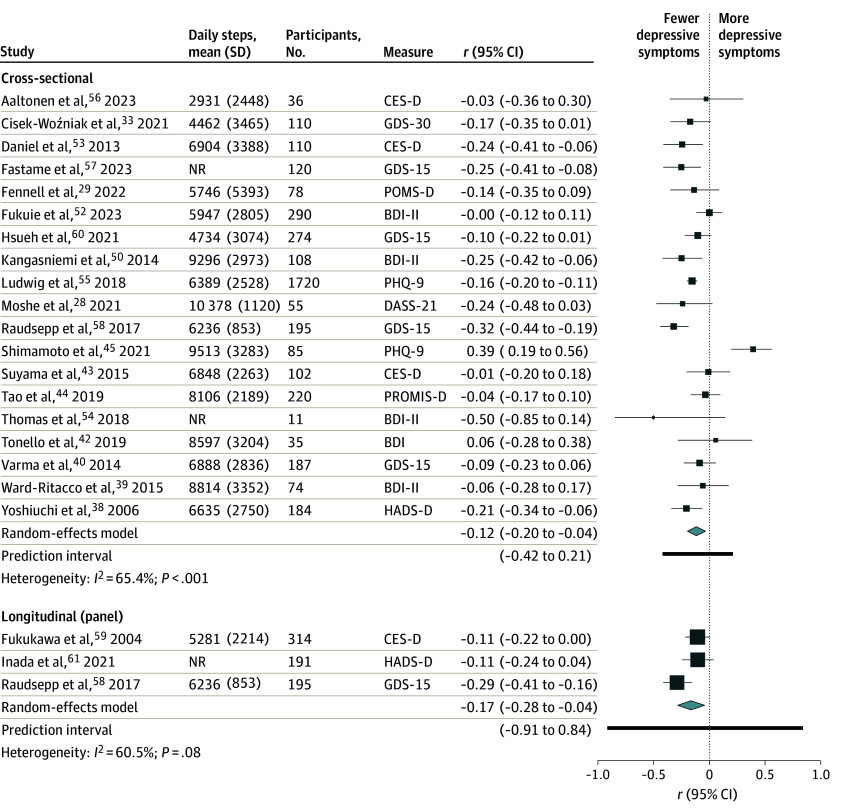
Pooled Correlation Between Higher Numbers of Daily Steps and Depressive Symptoms BDI indicates Beck Depression Inventory Scale; BDI-II, Beck Depression Inventory Scale, version 2; CES-D, Center for Epidemiologic Studies–Depression subscale; DASS-21, Depression Anxiety Stress Scale–21; GDS-15, 15-item Geriatric Depression Scale; GDS-30, 30-item Geriatric Depression Scale; HADS-D, Hospital Anxiety and Depression Scale–Depression subscale; NR, not reported; PHQ-9, Patient Health Questionnaire–9; POMS-D, Profile of Mood States–Depression scale; PROMIS-D, Patient-Reported Outcomes Measurement Information System–Depression scale.

A significant inverse correlation between the number of daily steps and depressive symptoms was identified in cross-sectional (*r*, −0.12; 95% CI, −0.20 to −0.04; *I*^2^ = 65.4%; n = 19^28,29,33,38,39,40,42,43,44,45,50,52,53,54,55,56,57,58,60^) and panel (*r*, −0.17; 95% CI, −0.28 to −0.04; *I*^2^ = 60.5%; n = 3^58,59,61^) studies (Figure 2). Furthermore, an increase of 1000 steps/d was associated with a significant reduction in the incidence of depression (RR, 0.91; 95% CI, 0.87-0.94; *I*^2^ = 48.3%; n = 2^62,63^) in prospective cohort studies (eTable 7 in Supplement 1).

### Daily Step Categories

**Figure 3.**
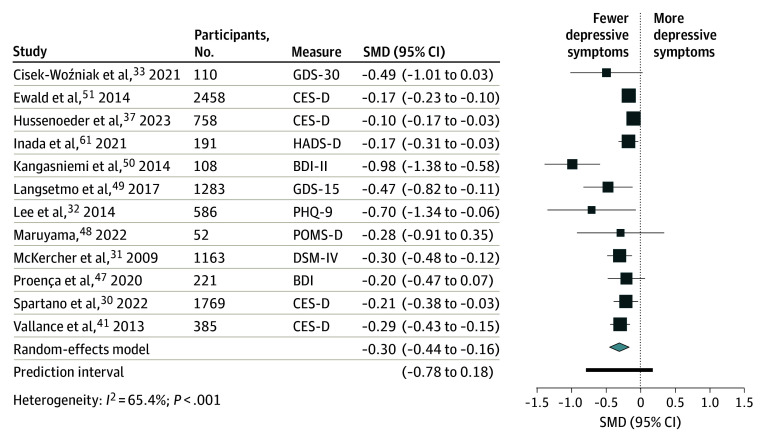
Pooled Standardized Mean Differences (SMDs) Between High (≥7500) vs Low (<7500) Numbers of Daily Steps and Depressive Symptoms BDI indicates Beck Depression Inventory Scale; BDI-II, Beck Depression Inventory Scale, version 2; CES-D, Center for Epidemiologic Studies–Depression subscale; DSM-IV, *Diagnostic and Statistical Manual of Mental Disorders*, 4th edition; GDS-15, 15-item Geriatric Depression Scale; GDS-30, 30-item Geriatric Depression Scale; HADS-D, Hospital Anxiety and Depression Scale–Depression subscale; PHQ-9, Patient Health Questionnaire–9; POMS-D, Profile of Mood States–Depression scale.

**Figure 4.**
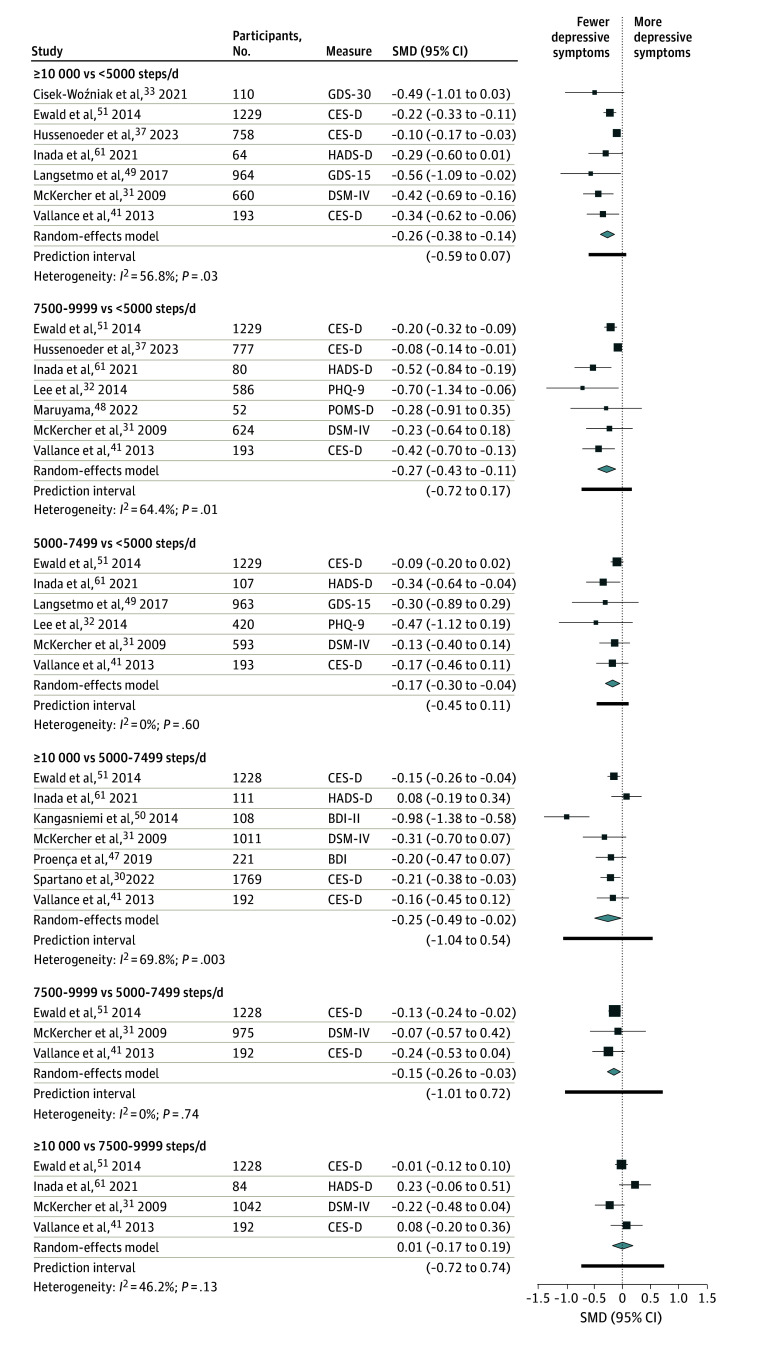
Pooled Standardized Mean Differences (SMDs) Between Daily Step Categories and Depressive Symptoms BDI indicates Beck Depression Inventory Scale; BDI-II, Beck Depression Inventory Scale, version 2; CES-D, Center for Epidemiologic Studies–Depression subscale; DSM-IV, *Diagnostic and Statistical Manual of Mental Disorders*, 4th edition; GDS-15, 15-item Geriatric Depression Scale; GDS-30, 30-item Geriatric Depression Scale; HADS-D, Hospital Anxiety and Depression Scale–Depression subscale; PHQ-9, Patient Health Questionnaire–9; POMS-D, Profile of Mood States–Depression scale.

Considering cross-sectional analyses, high (≥7500) vs low (<7500) counts of daily steps were associated with fewer depressive symptoms (SMD, −0.30; 95% CI, −0.44 to −0.16; *I*^2^ = 65.4%; n = 12^30,31,32,33,37,41,47,48,49,50,51,61^) in adults of all ages (Figure 3). Compared with the sedentary lifestyle category (<5000 steps/d), the pooled SMDs (Figure 4) revealed that higher counts of daily steps were associated with fewer depressive symptoms as follows: for active to highly active adults (≥10 000 steps/d), the SMD was −0.26 (95% CI, −0.38 to −0.14; *I*^2^ = 56.8%; n = 7^31,33,37,41,49,51,61^); for somewhat active adults (7500-9999 steps/d), the SMD was −0.27 (95% CI, −0.43 to −0.11; *I*^2^ = 64.4%; n = 7^31,32,37,41,48,51,61^); and for adults with low activity (5000-7499 steps/d), the SMD was −0.17 (95% CI, −0.30 to −0.04; *I*^2^ = 0%; n = 6^31,32,41,49,51,61^). When considering prospective cohort studies (eTable 7 in Supplement 1), adults with 7000 or more steps/d had a lower risk of depression than their counterparts with fewer than 7000 steps/d (RR, 0.69; 95% CI, 0.62-0.77; *I*^2^ = 0%; n = 2^60,62^).

### Subgroup Analyses and Meta-Regressions

Subgroup analyses are displayed in eTable 8 in Supplement 1. The pooled correlation estimate remained statistically significant for adults aged 36 to 64 years or 65 years or older, for step counters worn on the wrist, and for triaxial accelerations. Additionally, high (≥7500) vs low (<7500) counts of daily steps were significantly associated with fewer depressive symptoms in most cross-sectional subgroups, including all age groups, females and males, good- and fair-quality studies, and assessments using accelerometers and pedometers worn at the waist with uniaxial and triaxial accelerations. No statistically significant differences were identified within the categories in any of the subgroup analyses. Meta-regression models showed that none of the participant characteristics considered (age, sex, or BMI) significantly influenced the association between daily steps and depressive symptoms in cross-sectional studies (eTable 9 and eFigure 1 in Supplement 1).

### Sensitivity Analyses and Publication Bias

The pooled correlation coefficients and SMDs between daily steps and depressive symptoms remained significant when the leave-one-out method was used (eFigures 2 and 3 in Supplement 1). After studies that examined data specifically during COVID-19 lockdown were excluded,^28,29^ the inverse correlation remained (eTable 10 in Supplement 1). In addition, high (≥7500) vs low (<7500) counts of daily steps were associated with a decrease in depression as a categorical outcome in cross-sectional studies (OR, 0.58; 95% CI, 0.39-0.86; *I*^2^ = 0%; n = 4^30,31,32,33^) (eTable 10 in Supplement 1). According to the Egger test and funnel plot asymmetry (eTable 11 and eFigure 4 in Supplement 1), there was publication bias for the study association found from cross-sectional analysis comparing high (≥7500) vs low (<7500) counts of daily steps.

## Discussion

This systematic review and meta-analysis was, to our knowledge, the first to synthesize the associations between objectively measured daily steps and depression in the general adult population. The results of our cross-sectional analysis indicated that an increased number of daily steps was associated with a reduction in depressive symptoms. Compared with fewer than 5000 steps/d, reaching 5000 or more steps/d was associated with fewer depressive symptoms. In addition, counts above 7500 steps/d were associated with a 42% lower prevalence of depression. These findings corroborate evidence from cohort studies indicating that adults who increased their activity by 1000 steps/d and accumulated over 7000 steps/d had a 9% and 31% lower risk of developing depression, respectively.^60,62,63^

Our findings are consistent with those from a recent meta-analysis that suggested that small doses of PA were associated with a decreased incidence of depression in adults.^6^ However, the data were derived from studies in which PA measures were self-reported and estimated in marginal metabolic equivalent task hours per week,^6^ a measure that may not be readily understandable for the general population. The results of our meta-analysis indicated that increasing the number of daily steps, even at modest levels, was associated with a reduction in depressive symptoms. These results support a linear relationship within the range of daily steps examined in the included studies up to 10 000 steps/d. Beyond this range, as suggested for all-cause mortality,^14^ increasing the number of steps may not be associated with a significant reduction in depressive symptoms. However, this does not contradict the message that something is better than nothing,^64^ because as seen with depression and other health outcomes,^14^ even low PA levels showed protective associations.

Small amounts of PA may be particularly relevant for specific populations, such as older adults and individuals with limited activities of daily living, for whom daily steps emerge as an accessible PA strategy.^65^ The daily-steps approach has the potential to improve communication, adherence, feedback, prescribing, and self-monitoring with regard to PA levels.^10^ The use of simple activity monitors that allow continuous self-monitoring^66^ and the incorporation of specific goals^67^ have been associated with increasing daily steps in the general adult population. Therefore, setting goals for the number of daily steps may be a promising and inclusive public health strategy for the prevention of depression.

Although our findings suggest a protective association of increasing daily steps with depressive symptoms, it cannot yet be established whether there is a ceiling limit above which there would be no additional benefit from increasing steps. Furthermore, our study did not discriminate what proportion of this association was attributed to the type of PA and to other potential PA-related benefits, such as social well-being. Importantly, the optimal number of steps to prevent depression may vary according to sex, age, and individual risk of mental disorders.^31,62^ Thus, while the current study synthesizing evidence from observational studies suggests the potential for depression prevention, which may be achieved at certain ranges of step counts, specifically designed experimental studies are still needed to explore whether there are optimal and maximal step counts for specific population subgroups.

Our findings should be considered in the context of previous evidence regarding the PA characteristics that are most beneficial for the prevention of depression. A 2023 umbrella review revealed that all PA modalities (aerobic, strength, mixed mode [aerobic plus strength], and mind-body [qigong, stretching, tai chi, or yoga]) were associated with reduced depressive symptoms in both clinical and nonclinical populations.^68^ These findings are consistent with the results of a network meta-analysis comparing various structured exercise modalities (walking, running, dance, mixed aerobic practices, strength, qigong, tai chi, and yoga) in individuals with depression.^69^ However, other PA characteristics may yield mixed results in terms of their effectiveness in reducing depressive symptoms depending on the specific population studied.^68,69,70^ Moreover, additional elements of PA practices, such as the natural vs urban environment, may exert different influences on depressive symptoms.^71^

Different potential mechanisms have been proposed to explain the association between PA and depression, such as biological (inflammatory changes, mesolimbic pathway activation, neuroplasticity, and regulation of the hypothalamic-pituitary-adrenal axis) and psychosocial (self-efficacy, self-esteem, sleep quality, and social support) mechanisms.^72^ Regardless of the combination of mechanisms responsible for associations between PA and prevention of depression, a daily active lifestyle may be a crucial factor in regulating and reinforcing these pathways.^8^

### Strengths and Limitations

Several strengths must be mentioned. First, our findings provide new insights to guide recommendations for promoting step-based PA goals in depression prevention. Second, compared with self-reported measures, the use of objective PA measures could more accurately reflect the associations between daily steps and depression. Third, the large adult population included in our analysis was geographically diverse and provided a global picture of the study associations. Fourth, our study shed light on the limitations of the available evidence and provided some recommendations to improve the quality and comparability of future studies on the association between daily steps and depression (eTable 12 in Supplement 1).

Some limitations must also be acknowledged. First, reverse associations are possible, and causal inferences cannot be made. Second, our indicators showed substantial between-study heterogeneity in some pooled estimates that could be partially explained by differences in participant characteristics (age, sex) and step-counting devices (type, position, and acceleration axis). Third, we cannot rule out residual confounding due to factors unavailable in most of the studies, such as PA modalities without steps or chronic stress. Fourth, most studies lacked robust methods, which may have affected the reliability of the results. Thus, although the findings were not significant, the subgroup analysis restricted to high-quality studies revealed a decrease in the pooled estimate and heterogeneity levels in comparison with studies of fair quality. Fifth, the meta-analysis from cross-sectional studies comparing high vs low counts of daily steps might have been susceptible to publication bias, which could have influenced the overall estimate of the study association. Sixth, the 95% prediction interval results indicate that the conclusions of this study should be treated with caution given the uncertainty of the results.

## Conclusions

This systematic review and meta-analysis synthesized, for the first time to our knowledge, the associations between objectively measured daily steps and depression in the general adult population. Our results showed significant associations between higher numbers of daily steps and fewer depressive symptoms as well as lower prevalence and risk of depression in the general adult population. The objective measurement of daily steps may represent an inclusive and comprehensive approach to public health that has the potential to prevent depression. We underline the need for further cohort studies to clarify the potential protective role of daily steps in mitigating the risk of depression during adulthood.

## Notes

Supplement 1. **eAppendix.** Supplementary Methods **eTable 1.** Detailed Information on Search Strategies **eTable 2.** Effect Sizes, Main Results, and Covariate Adjustments of Included Studies **eTable 3.** List of the Studies Fully Assessed for Eligibility and Excluded **eTable 4.** Characteristics of the Included Studies **eTable 5.** Characteristics of the Populations, Exposures, and Outcomes of the Included Studies **eTable 6.** Quality Assessment of Included Studies **eTable 7.** Daily Steps and Risk of Depression in Prospective Cohort Studies **eTable 8.** Subgroup Analyses by Age, Sex, Step Counter Characteristics, and Study Quality **eTable 9.** Meta-Regression Analyses by Age, Sex, and Body Mass Index **eTable 10.** Sensitivity Analyses **eTable 11.** Publication Bias **eTable 12.** Recommendations for Future Studies on the Associations Between Objectively Measured Daily Steps and Depression in the General Adult Population **eFigure 1.** Meta-Regression Models by Age, Sex, and Body Mass Index **eFigure 2.** Sensitivity Analysis by Leave-One-Out Method in Pooled Correlation Coefficients of Cross-Sectional Studies **eFigure 3.** Sensitivity Analysis by Leave-One-Out Method in Pooled Standardized Mean Differences of Cross-Sectional Studies **eFigure 4.** Publication Bias Funnel Plots

Supplement 2.
Data Sharing Statement
