# Supplementary material for: Daily Step Count and Depression in Adults: A Systematic Review and Meta-Analysis
Source: JAMA Netw Open. 2024 Dec 16;7(12):e2451208. doi: 10.1001/jamanetworkopen.2024.51208 (PMC11650418; doi:10.1001/jamanetworkopen.2024.51208)
Supplement: Supplement 1. — eAppendix. Supplementary Methods eTable 1. Detailed Information on Search Strategies eTable 2. Effect Sizes, Main Results, and Covariate Adjustments of Included Studies eTable 3. List of the Studies Fully Assessed for Eligibility and Excluded eTable 4. Characteristics of the Included Studies eTable 5. Characteristics of the Populations, Exposures, and Outcomes of the Included Studies eTable 6. Quality Assessment of Included Studies eTable 7. Daily Steps and Risk of Depression in Prospective Cohort Studies eTable 8. Subgroup Analyses by Age, Sex, Step Counter Characteristics, and Study Quality eTable 9. Meta-Regression Analyses by Age, Sex, and Body Mass Index eTable 10. Sensitivity Analyses eTable 11. Publication Bias eTable 12. Recommendations for Future Studies on the Associations Between Objectively Measured Daily Steps and Depression in the General Adult Population eFigure 1. Meta-Regression Models by Age, Sex, and Body Mass Index eFigure 2. Sensitivity Analysis by Leave-One-Out Method in Pooled Correlation Coefficients of Cross-Sectional Studies eFigure 3. Sensitivity Analysis by Leave-One-Out Method in Pooled Standardized Mean Differences of Cross-Sectional Studies eFigure 4. Publication Bias Funnel Plots [file jamanetwopen-e2451208-s001.pdf]

Supplementary Online Content

Bizzozero-Peroni B, Díaz-Goñi V, Jiménez-López E, et al. Daily step count and depression in adults: a systematic review and meta-analysis. *JAMA Netw Open*. 2024;7(12):e2451208. doi:10.1001/jamanetworkopen.2024.51208

eAppendix. Supplementary Methods
eTable 1. Detailed Information on Search Strategies
eTable 2. Effect Sizes, Main Results, and Covariate Adjustments of Included Studies
eTable 3. List of the Studies Fully Assessed for Eligibility and Excluded
eTable 4. Characteristics of the Included Studies
eTable 5. Characteristics of the Populations, Exposures, and Outcomes of the Included Studies
eTable 6. Quality Assessment of Included Studies
eTable 7. Daily Steps and Risk of Depression in Prospective Cohort Studies
eTable 8. Subgroup Analyses by Age, Sex, Step Counter Characteristics, and Study Quality
eTable 9. Meta-Regression Analyses by Age, Sex, and Body Mass Index
eTable 10. Sensitivity Analyses
eTable 11. Publication Bias
eTable 12. Recommendations for Future Studies on the Associations Between Objectively Measured Daily Steps and Depression in the General Adult Population
eFigure 1. Meta-Regression Models by Age, Sex, and Body Mass Index
eFigure 2. Sensitivity Analysis by Leave-One-Out Method in Pooled Correlation Coefficients of Cross-Sectional Studies
eFigure 3. Sensitivity Analysis by Leave-One-Out Method in Pooled Standardized Mean Differences of Cross-Sectional Studies
eFigure 4. Publication Bias Funnel Plots

This supplementary material has been provided by the authors to give readers additional information about their work.

© 2024 Bizzozero-Peroni B et al. *JAMA Network Open*.

eAppendix. Supplementary Methods.

Inclusion criteria: outcome

Depression, as a diagnosis or as depressive symptoms, including 1) depressive disorders determined by physicians or trained health professionals using recognized diagnostic schedules, registry data, or self-report of medical diagnosis, and 2) the severity of depressive symptoms (i.e., indicated by the number of symptoms or the presence of mild to severe symptoms) using validated observer rating or self-rating scales (overall score).

Exclusion criteria

Studies were excluded if they reported: 1) exclusive data on populations with other diseases or medical conditions (e.g., hospitalized patients, obesity) or with professions closely linked to exercise (e.g., elite athletes, military service); 2) exclusively self-reported data on daily steps; 3) objective step count data related to exercise or sport sessions only; 4) walking as exposure without daily step count; 5) depression data that could not be isolated and extracted (e.g., overall mood states, psychological stress) or specific items of self-reported scales; 6) depression related to pregnancy (i.e., peripartum or postpartum depression); 7) duplicated data published in another included study; and 8) noneligible publications such as conference or meeting abstracts, preprints, and letters to the editor.

Data extraction

The following data were extracted from the included studies: 1) authors and year of publication; 2) country where the data were collected; 3) study design and follow-up period; 4) sample size; 5) characteristics of study participants, including age, sex, body mass index (BMI), and type of population; 6) characteristics related to daily step count, including activity levels (mean and range), type of step-counting device, duration of monitoring period and validity of data (days and wear time required for analysis), wearing position of device, and exposure harmonization (higher number of daily steps and/or comparison across daily step categories); 7) measures of depression (diagnostic tools) or

© 2024 Bizzozero-Peroni B et al. *JAMA Network Open*.

depressive symptoms (assessment scales); 8) results on depression (percentage of individuals with depressive disorder or mild to severe depressive symptoms, or number of depressive symptoms); and 9) associations between daily step count and depression, including statistical methods, effect size estimations, and covariate adjustments.

Quality assessment

The National Institutes of Health (NIH)'s Quality Assessment Tool for Observational Cohort and Cross-Sectional Studies was used to evaluate the methodological quality of the observational studies.[1] This tool evaluates 14 items in domains such as study population, timeframe for associations, exposure and outcome measures, or statistical analyses. Each criterion could be scored as "yes" when the study achieved the criterion, "no" when the study did not achieve the criterion, and "not reported" when the studies did not clearly report the required information. The answers to these domains contributed to an overall judgement. The quality of each cross-sectional and longitudinal study was rated as good (if most criteria were met), fair (if some criteria were met), or poor (if few criteria were met) according to the NIH quality rating guide (**eTable 6**).

Exposure harmonization

We harmonized the comparisons reported in the included studies into two groups according to the 2011 American College of Sports Medicine position statement on minimum daily steps (7000–8000 steps/day) for health benefits.[2] Two different cutoff points were used according to the data provided by the included studies (7500 or 7000 steps/day for cross-sectional or prospective cohort studies, respectively). Therefore, we categorized two groups of daily steps as follows: high (≥7500 or ≥7000 steps/day) and low (<7500 or <7000 steps/day) levels. This free-living PA threshold (7000–8000 steps/day) for healthy adults is associated with public health guidelines' emphasis on minimal amounts of time spent in moderate to vigorous PA[3] and with recent evidence on the optimal dose for reducing the risk of all-cause mortality.[4] In turn, we operationalized daily step categories

© 2024 Bizzozero-Peroni B et al. *JAMA Network Open*.

into the graduated step index proposed by Tudor-Locke et al.[5] to estimate habitual PA in adults: 1) ≥10 000 steps/day (active to highly active), 2) 7500–9999 steps/day (somewhat active), 3) 5000–7499 steps/day (low active), and 4) <5000 steps/day (sedentary).

Some considerations of exposure data collection should be detailed. The PA levels of the included studies were grouped into the above categories according to the mean or range of daily steps. In those cases where a study reported the exposure categories as ranges different from those proposed by Tudor-Locke et al.,[5] the midpoint of the upper and lower limits was considered to define the daily step category.[6,7] For open categories, the midpoint of the category was assumed to be the width of the adjacent category.[8] Furthermore, in studies that provided results on depression according to daily step categories[7] or dose–response analysis[9] only graphically, data were extracted using WebPlotDigitizer software.[10] For the dose–response curve, the midpoint of each daily step category[5] was considered to extract the results on depression (mean, upper and lower limits).

Effect sizes

Since most cross-sectional and longitudinal panel studies have analyzed the correlations between a higher number of daily steps (continuous variable) and depressive symptoms, the pooled correlation coefficients were estimated. Negative values of *r* and standardized mean differences (SMDs) indicate fewer depressive symptoms in favor of a higher number of daily steps. The criterion proposed by Cohen to classify the effect size estimators as small ($r \approx 0.10$, SMD≈0.20), medium ($r \approx 0.30$, SMD≈0.50) or large ($r \approx 0.50$, SMD≈0.80) was considered. Odds ratio and risk ratio values less than 1 indicate a lower prevalence or risk of depression in favor of a higher number of daily steps.

Data synthesis

A series of meta-analyses were performed with the objective of estimating the following associations: (i) cross-sectional and (ii) longitudinal (panel studies) correlations between

© 2024 Bizzozero-Peroni B et al. *JAMA Network Open*.

daily steps and depressive symptoms (**Figure 2**); (iii) high *vs*. low levels of daily steps and cross-sectional SMDs in depressive symptoms (**Figures 3–4**); (iv) high *vs*. low levels of daily steps and odds of depression or mild-to-severe depressive symptoms in cross-sectional studies (**eTable 10**); and (v) an increase of 1000 steps/day or high *vs*. low levels of daily steps and the risk of depression or mild-to-severe depressive symptoms in prospective cohort studies (**eTable7**).

Methodological considerations

Other methodological considerations of data collection and analysis should be noted. If the included studies displayed adjustment models, those that reflected the maximum extent of adjustment were selected. When studies reported exposure harmonization as continuous and categorical variables,[11–14] data were included according to the appropriate meta-analysis. For studies with different categories of daily steps (**eTable2**),[6–9,15–17] data were included according to the appropriate comparison group on the classification system proposed by Tudor-Locke et al.[5] (**Figure 4**).

When studies reported two or more results on depression for the same category of daily steps (**eTable2**),[7,9,15–17] we combined the respective measures to calculate a single pooled SMD for meta-analyses comparing high *vs*. low levels of daily steps (**Figures 3–4**). In those cases where studies stratified the results by sex[9,17] and where >1 cross-sectional result was reported,[15,18,19] we combined the respective measures to calculate a single pooled estimate for each study. For each of these combinations, we performed a three-level random effects meta-analysis.[20] The multilevel approach accounts for dependence between effect sizes and includes three pooling steps (i.e., sampling variance, variance between effect sizes from the same sample, and variance between studies) that result in an overall true effect size, increasing statistical power, and extracting maximum information from the data.[21] The estimate and standard error for each multilevel analysis were extracted and subsequently applied to the main meta-analyses.

© 2024 Bizzozero-Peroni B et al. *JAMA Network Open*.

For cohort studies, hazard ratios,[12,22] odds ratios,[12] and rate ratios[11] were considered equivalent to risk ratio because the population rates of incident cases related to depression were low (i.e., <10%).[23–25] Similarly, prevalence ratios[17] and rate ratios[6] employed in cross-sectional studies were deemed to be equivalent to odds ratios. In two longitudinal studies with >1 follow-up length,[18] we considered the longest period. In a prospective cohort study that reported both univariate and multivariate estimates, as well as both unstandardized and standardized data, we selected the multivariate, standardized data.[22] Finally, longitudinal studies reporting baseline results on the proposed association were included in the cross-sectional analyses.[11,15,18]

Dealing with missing data

Corresponding authors of seven studies[6,8,11,15,18,26,27] were contacted at least twice by e-mail or the ResearchGate social networking site to request substantial missing data for the study's association (i.e., mean or range of daily steps, percentage of individuals with mild to severe depressive symptoms). Three studies[6,8,11] provided additional data of interest that contributed to the present analyses. When authors did not send additional data, we reported them as missing values.

References

1. National Heart Lung and Blood Institute. Quality Assessment Tool for Observational Cohort and Cross-sectional Studies. Published 2021. https://www.nhlbi.nih.gov/health-topics/study-quality-assessment-tools (accessed 20 May 2024).

2. Garber CE, Blissmer B, Deschenes MR, et al. Quantity and quality of exercise for developing and maintaining cardiorespiratory, musculoskeletal, and neuromotor fitness in apparently healthy adults: Guidance for prescribing exercise. *Med Sci Sports Exerc*. 2011;43(7). doi: 10.1249/MSS.0b013e318213fefb.

3. Tudor-Locke C, Craig CL, Brown WJ, et al. How many steps/day are enough? for adults. *Int J Behav Nutr Phys Act*. 2011;8(79). doi: 10.1186/1479-5868-8-79.

© 2024 Bizzozero-Peroni B et al. *JAMA Network Open*.

4. Paluch AE, Gabriel KP, Fulton JE, et al. Steps per Day and All-Cause Mortality in Middle-aged Adults in the Coronary Artery Risk Development in Young Adults Study. *JAMA Netw Open*. 2021;4(9). doi: 10.1001/jamanetworkopen.2021.24516.

5. Tudor-Locke C, Hatano Y, Pangrazi RP, et al. Revisiting "how many steps are enough?" *Med Sci Sports Exerc*. 2008;40(7 Suppl). doi: 10.1249/MSS.0b013e31817c7133.

6. Hussenoeder FS, Conrad I, Pabst A, et al. Physical activity and mental health: the connection between step count and depression, anxiety and quality of sleep. *Psychol Health Med*. 2022. doi: 10.1080/13548506.2022.2159453.

7. Vallance JK, Eurich D, Lavallee C, et al. Daily Pedometer Steps among Older Men: Associations with Health-Related Quality of Life and Psychosocial Health. *Am J Health Promot*. 2013;27(5). doi: 10.4278/ajhp.120316-QUAN-145.

8. Lee H, Lee JA, Brar JS, *et al*. Physical activity and depressive symptoms in older adults. *Geriatr Nurs*. 2014;35(1). doi: 10.1016/j.gerinurse.2013.09.005.

9. Ewald B, Attia J, McElduff P. How Many Steps Are Enough? Dose–Response Curves for Pedometer Steps and Multiple Health Markers in a Community-Based Sample of Older Australians. *J Phys Act Health*. 2014;11(3). doi: 10.1123/jpah.2012-0091.

10. Rohatgi A. WebPlotDigitizer 4.6. Published 2022. https://automeris.io/WebPlotDigitizer (Accessed 15 July 2023).

11. Hsueh MC, Stubbs B, Lai YJ, et al. A dose response relationship between accelerometer assessed daily steps and depressive symptoms in older adults: a two-year cohort study. *Age Ageing*. 2021;50(2). doi: 10.1093/ageing/afaa162.

12. Master H, Annis J, Huang S, et al. Association of step counts over time with the risk of chronic disease in the All of Us Research Program. *Nat Med*. 2022;28(11). doi: 10.1038/s41591-022-02012-w.

© 2024 Bizzozero-Peroni B et al. *JAMA Network Open*.

13. Cisek-Woźniak A, Mruczyk K, Wójciak RW. The Association between Physical Activity and Selected Parameters of Psychological Status and Dementia in Older Women. *Int J Environ Res Public Health*. 2021;18(14). doi: 10.3390/ijerph18147549.
14. Kangasniemi A, Lappalainen R, Kankaanpää A, et al. Mindfulness skills, psychological flexibility, and psychological symptoms among physically less active and active adults. *Ment Health Phys Act*. 2014;7(3). doi: 10.1016/j.mhpa.2014.06.005.
15. Inada S, Yoshiuchi K, Park S, et al. Trajectories of objectively measured physical activity and mood states in older Japanese adults: longitudinal data from the Nakanojo Study. *Biopsychosoc Med*. 2021;15(1). doi: 10.1186/s13030-021-00207-0.
16. Langsetmo L, Kats AM, Cawthon PM, et al. The Association Between Objectively Measured Physical Activity and Subsequent Health Care Utilization in Older Men. *J Gerontol A Biol Sci Med Sci*. 2019;74(6):820-826. doi: 10.1093/GERONA/GLX191.
17. McKercher CM, Schmidt MD, Sanderson KA, et al. Physical Activity and Depression in Young Adults. *Am J Prev Med*. 2009;36(2). doi: 10.1016/j.amepre.2008.09.036.
18. Raudsepp L, Riso EM. Longitudinal Association Between Objectively Measured Walking and Depressive Symptoms Among Estonian Older Adults. *J Aging Phys Act*. 2017;25(4). doi: 10.1123/japa.2016-0303.
19. Fennell C, Eremus T, Puyana MG, et al. The Importance of Physical Activity to Augment Mood during COVID-19 Lockdown. *Int J Environ Res Public Health*. 2022;19(3). doi: 10.3390/ijerph19031270.
20. Cheung MW. Modeling dependent effect sizes with three-level meta-analyses: a structural equation modeling approach. *Psychol Methods*. 2014;19(2):211-29. doi: 10.1037/a0032968.
21. Van den Noortgate W, López-López JA, Marín-Martínez F, Sánchez-Meca J. Three-level meta-analysis of dependent effect sizes. *Behav Res Methods*. 2013;45(2):576-94. doi: 10.3758/s13428-012-0261-6.

© 2024 Bizzozero-Peroni B et al. *JAMA Network Open*.

22. Chan LLY, Brodie MA, Lord SR. Prediction of Incident Depression in Middle-Aged and Older Adults using Digital Gait Biomarkers Extracted from Large-Scale Wrist Sensor Data. *J Am Med Dir Assoc*. 2023;24(8):1106-1113.e11. doi: 10.1016/J.JAMDA.2023.04.008

23. Norton EC, Dowd BE, Maciejewski ML. Odds Ratios-Current Best Practice and Use. JAMA. 2018 Jul 3;320(1):84-85. doi: 10.1001/jama.2018.6971.

24. Higgins JPT, Thomas J, Chandler J, Cumpston M, Li T, Page MJ, et al. Cochrane Handbook for Systematic Reviews of Interventions. Chichester: John Wiley & Sons, 2019.

25. Greenland S. Interpretation and choice of effect measures in epidemiologic analyses. *Am J Epidemiol*. 1987;125(5):761-8. doi: 10.1093/oxfordjournals.aje.a114593.

26. Fukukawa Y, Nakashima C, Tsuboi S, et al. Age differences in the effect of physical activity on depressive symptoms. *Psychol Aging*. 2004;19(2). doi: 10.1037/0882-7974.19.2.346.

27. Varma VR, Tan EJ, Wang T, et al. Low-Intensity Walking Activity Is Associated With Better Health. *J Appl Gerontol*. 2013;33(7). doi: 10.1177/0733464813512896.

eTable 1. Detailed information on search strategies.

Information source	Method
Databases:[a] PubMed,[b] PsychINFO,[b] Scopus,[c] SPORTDiscus,[b] Web of Science[c]	(step* OR walk* OR gait OR training OR exercise OR "physical activity") AND (wearable OR pedometer* OR acceleromet* OR "movement counter" OR "activity monitor" OR "activity band" OR "activity bracelet" OR "activity technology" OR "activity tracker" OR "quantitative measurement" OR "objective measurement" OR "quantified movement" OR fitbit OR actigraph*) AND (depress* OR mood OR "affective disorder" OR dysthymi* OR psychologic*).
Online resource and browsing	Portal (URL): Google Scholar (https://scholar.google.com). Search terms: “daily steps” AND “depression” AND “adults”. The first 100 records (n=10 pages) were selected and screened.
Citation searching	Relevant systematic reviews were kept separate to screen their included studies.[1–3] References of included articles were manually screened to identify additional studies.
Other	PubMed’s similar articles search (first 5 records) was performed on all included articles.

[a] Data were retained in Research Information Systems (RIS) or PubMed formats for each database. [b] Search strategy restricted to Title/Abstract. [c] Search strategy restricted to Title/Abstract/Keywords.

References cited in this table:

1. Gianfredi V, Blandi L, Cacitti S, Minelli M, Signorelli C, Amerio A, et al. Depression and Objectively Measured Physical Activity: A Systematic Review and Meta-Analysis. *Int J Environ Res Public Health.* 2020;17(10):3738. doi: 10.3390/ijerph17103738.

2. Aoyagi Y, Shephard RJ. Sex differences in relationships between habitual physical activity and health in the elderly: practical implications for epidemiologists based on pedometer/accelerometer data from the Nakanojo Study. *Arch Gerontol Geriatr*. 2013;56(2):327–38. doi: 10.1016/j.archger.2012.11.006.

3. Bourke M, Patten RK, Klamert L, Klepac B, Dash S, Pascoe MC. The acute affective response to physical activity in people with depression: A meta-analysis. *J Affect Disord*. 2022;311:353–63. doi: 10.1016/j.jad.2022.05.089.

© 2024 Bizzozero-Peroni B et al. *JAMA Network Open*.

eTable 2. Effect sizes, main results, and covariate adjustments of included studies.

Reference	Exposure harmonization (daily steps)	Data	Depression outcome (measure)	Effect size	Result	Covariate adjustment
Aaltonen et al.[56] 2023	Increase	Cross-sectional	Symptoms (CES–D)	Pearson's *r* (*p*-value)	−0.030 (>0.050)	NA
Chan et al.[63] 2023	Increasing 1000 steps	Longitudinal (prospective cohort)	Depressive episode (hospital inpatient data, ICD–10)	HR (95% CI)	0.88 (0.82−0.93)	Age, sex, BMI, marital status, household income, education level, mode of transportation, smoking and drinking status, presence of abnormal sleeping duration, alternated sleep phase, and severe medical conditions
Cisek-Wozniak et al.[33] 2021	Increase	Cross-sectional	Symptoms (GDS–30)	Spearman's *r* (*p*-value)	−0.165 (0.844)	NA
	High (11 335) *vs*. low (3205)			Mean (SD)	2.5 (2.5) *vs*. 4.1 (3.4)	
	High (11 335) *vs*. low (3205)			Nonevents/events	17/0 *vs*. 86/7	
Daniel et al.[53] 2013	Increase	Cross-sectional	Symptoms (CES–D)	Pearson's *r* (*p*-value)	−0.244 (>0.050)	NA
Ewald et al.[51] 2014	High (12 465) *vs*. low (3500)	Cross-sectional	Symptoms (CES–D)	Mean (SD), men / women	5.9 (8.5) *vs*. 7.9 (8.0) / 7.1 (7.5) *vs*. 8.5 (7.2)	Age, smoking status, household income, education level, alcohol consumption
	High (8 750) *vs*. low (3500)				6.0 (8.6) *vs*. 7.9 (8.0) / 7.2 (7.6) *vs*. 8.5 (7.2)	
	High (6 250) *vs*. low (3500)				7.3 (7.0) *vs*. 7.9 (8.0) / 7.9 (5.7) *vs*. 8.5 (7.2)	
	High (12 465) *vs*. low (6250)				5.9 (8.5) *vs*. 7.3 (7.0) / 7.1 (7.5) *vs*. 7.9 (5.7)	
	High (8750) *vs*. low (6250)				6.0 (8.6) *vs*. 6.0 (8.6) / 7.2 (7.6) *vs*. 7.9 (5.7)	
	High (12 465) *vs*. low (8750)				5.9 (8.5) *vs*. 6.0 (8.6) / 7.1 (7.5) *vs*. 7.2 (7.6)	
Fastame et al.[57] 2023	Increase	Cross-sectional	Symptoms (GDS–15)	Pearson's *r* (*p*-value)	−0.252 (<0.010)	NA
Fennell et al.[29] 2022	Increase	Cross-sectional	Symptoms (POMS–D)	β (*p*-value)	0.001 (0.994), −0.277 (0.014)	Age, sex, weight

© 2024 Bizzozero-Peroni B et al. *JAMA Network Open*.

Fukuie et al.[52] 2023	Increase	Cross-sectional	Symptoms (BDI–II)	β (*p*-value)	−0.00[a] (>0.050)	NA
Fukukawa et al.[59] 2004[b]	Increase	Longitudinal (panel study)	Symptoms (CES–D)	β (*p*-value)	−0.110 (<0.050)	Sex, annual family incomes, chronic conditions
Hsueh et al.[60] 2021	Increasing 1000 steps	Cross-sectional	Symptoms (GDS–15)	Spearman's *r* (*p*-value)	−0.100 (>.05)	Accelerometer wear time
	High (≥7000) *vs*. low (<3500)	Longitudinal (prospective cohort)		RR (95% CI)	0.66 (0.49−0.89)	Age, sex, chronic diseases, ADL, depressive symptoms (baseline), education level, accelerometer wear time
Hussenoeder et al.[37] 2022	High (≥10 000) *vs*. low (<5000)	Cross-sectional	Symptoms (CES–D)	RR (95% CI)	0.84 (0.74−0.95)	Age, sex, marital status, living situation, education, medical history, household, income, occupational status, personality
	High (5000–9999) *vs*. low (<5000)				0.87 (0.77−0.98)	
Inada et al.[61] 2021	High (10 812) *vs*. low (3811)	Cross-sectional	Symptoms (HADS–D)	Mean (SD)	4.7 (2.9), 4.6 (3.3), 7.5 (2.1) *vs*. 5.5 (3.2); 3.5 (2.5), 3.7 (1.8), 4.5 (3.5) *vs*. 4.9 (3.5)	NA
	High (8207) *vs*. low (3811)				3.8 (2.9) *vs*. 5.5 (3.2); 3.4 (3.0) *vs*. 4.9 (3.5)	
	High (6090) *vs*. low (3811)				4.0 (3.9) *vs*. 5.5 (3.2); 3.9 (3.8) *vs*. 4.9 (3.5)	
	High (10 812) *vs*. low (6090)				4.7 (2.9), 4.6 (3.3), 7.5 (2.1) *vs*. 4.0 (3.9); 3.5 (2.5), 3.7 (1.8), 4.5 (3.5) *vs*. 4.0 (3.9)	
	High (10 812) *vs*. low (8207)				4.7 (2.9), 4.6 (3.3), 7.5 (2.1) *vs*. 3.8 (2.9); 3.5 (2.5), 3.7 (1.8), 4.5 (3.5) *vs*. 3.4 (3.0)	
	High (≥8000) *vs*. low (<7000)	Longitudinal (panel study)		Mean difference (SE)	−0.4 (0.6), −1.2 (0.7), −0.9 (1.5), −3.0 (1.8) *vs*. −0.6 (0.9), −0.1 (0.6)	Age, depressive symptoms (baseline)
Kangasniemi et al.[50] 2014	Increase	Cross-sectional	Symptoms (BDI–II)	Pearson's *r* (*p*-value)	−0.250 (<.05)	NA
	High (12 050) *vs*. low (6921)			Mean (SD)	2.9 (3.4) *vs*. 7.4 (5.4)	BMI, health problems

© 2024 Bizzozero-Peroni B et al. *JAMA Network Open*.

Langsetmo et al.[49] 2017	High (13 199) *vs*. low (2694)	Cross-sectional	Symptoms (GDS–15)	Mean (SD)	1.1 (1.3) *vs*. 2.1 (2.0)	NA
	High (6466) *vs*. low (2694)				1.5 (1.5) *vs*. 2.1 (2.0)	
Lee et al.[32] 2014	High (≥7500) *vs*. low (<5000)	Cross-sectional	Symptoms (PHQ–9)	OR (95% CI)	0.28 (0.09−0.91)	Age, chronic conditions, annual household income
	High (5000–7499) *vs*. low (<5000)				0.43 (0.13−1.38)	
Ludwig et al.[55] 2018	Increase	Cross-sectional	Symptoms (PHQ–9)	Spearman's *r* (*p*-value)	−0.149 (.010)	NA
Maruyama.[48] 2022	High (9186) *vs*. low (3905)	Cross-sectional	Symptoms (POMS–D)	Mean (SD)	44.2 (5.6) *vs*. 45.8 (5.7)	NA
Master et al.[62] 2022	Increasing 1000 steps	Longitudinal (prospective cohort)	Major depression (hospital inpatient data, ICD–9)	OR (95% CI)	0.92 (0.89−0.95)	Age, sex, race
	High (10 720) *vs*. low (6090)			HR (95% CI)	0.70 (0.59−0.84)	Age, sex, race, blood pressure, coronary artery disease, cancer, smoking, education, alcohol, BMI, Fitbit wear time, step counts (mean of first 6 months)
Mckercher et al.[31] 2009	High (≥12 500) *vs*. low (<5000)	Cross-sectional	Major or persistent depression (diagnostic interview, DSM-IV)	PR (95% CI), men / women	0.48 (0.11−2.01) / 0.42 (0.17−1.05)	Age, smoking, income, BMI, marital status, education, physical health status
	High (10 000–12 499) *vs*. low (<5000)				0.72 (0.20−2.62) / 0.43 (0.21−0.87)	
	High (7500–9999) *vs*. low (<5000)				1.19 (0.36−3.88) / 0.52 (0.29−0.94)	
	High (5000–7499) *vs*. low (<5000)				0.94 (0.27−3.25) / 0.76 (0.45−1.30)	
	High (≥10 000) *vs*. low (5000–7499)			Nonevents/events	507/32 *vs*. 422/50	NA
	High (≥10 000) *vs*. low (7500–9999)				507/32 *vs*. 459/44	
	High (7500–9999) *vs*. low (5000–7499)				459/44 *vs*. 422/50	
Moshe et al.[28] 2021	Increase	Cross-sectional	Symptoms (DASS–21)	Spearman's *r* (*p*-value)	−0.230 (.52)	NA
Proenca et al.[47] 2020	High (10 287) *vs*. low (6546)	Cross-sectional	Symptoms (BDI)	Mean (SD)	3.7 (5.6) *vs*. 4.8 (5.4)	NA
Raudsepp and Riso.[58] 2017	Increase	Cross-sectional	Symptoms (GDS–15)	r^c (*p*-value)	−0.350, −0.290, −0.320 (<.001)	Age, sex, health status
		Longitudinal (panel study)			−0.290 (<.001)	NA

© 2024 Bizzozero-Peroni B et al. *JAMA Network Open*.

Sato et al.[46] 2021[d]	High (7182) *vs*. low (6000)	Cross-sectional	Symptoms (TQS)	OR (95% CI)	0.82 (0.69−0.97)	Age, sex, suspension or loss of work, working hours, work at home, time on childcare
Shimamoto et al.[45] 2021	Increase	Cross-sectional	Symptoms (PHQ–9)	*r*[c] (*p*-value)	0.390 (<.001)	NA
Spartano et al.[30] 2021	High (10 371) *vs*. low (6671)	Cross-sectional	Symptoms (CES–D)	Nonevents/events	845/73 *vs*. 756/95	NA
Suyama et al.[43] 2015	Increase	Cross-sectional	Symptoms (CES–D)	Pearson's *r* (*p*-value)	−0.010 (>.05)	NA
Tao et al.[44] 2019	Increase	Cross-sectional	Symptoms (PROMIS–D)	Pearson's *r* (*p*-value)	−0.036 (>.05)	NA
Thomas and Vejlgaard.[54] 2018	Increase	Cross-sectional	Symptoms (BDI-II)	Pearson's *r* (*p*-value)	−0.244 (<.05)	NA
Tonello et al.[42] 2019	Increase	Cross-sectional	Symptoms (BDI)	Pearson's *r* (*p*-value)	0.056 (>.05)	NA
Vallance et al.[41] 2013	High (≥11 392) *vs*. low (2785)	Cross-sectional	Symptoms (CES–D)	Mean difference (95% CI)	−2.65 (−4.96 to −0.34)	Age, household income, BMI, comorbidities (i.e., history of heart disease, stroke, diabetes, or cancer)
	High (9754) *vs*. low (2785)				−3.26 (−5.56 to −0.96)	
	High (6843) *vs*. low (2785)				−1.36 (−3.58 to 0.89)	
	High (16 854) *vs*. low (6843)				−1.29 (−0.45 to −0.17)	
	High (9754) *vs*. low (6843)				−1.90 (−0.53 to −0.24)	
Varma et al.[40] 2014	Increasing 1000 steps	Cross-sectional	Symptoms (GDS–15)	β (95% CI)	−0.09 (−0.17 to −0.01)	Age, sex, race
Ward-Ritacco et al.[39] 2014	Increase	Cross-sectional	Symptoms (BDI–II)	*r*[c] (*p*-value)	−0.060 (>.05)	NA
Yoshiuchi et al.[38] 2006	Increase	Cross-sectional	Symptoms (HADS–D)	*r*[c] (*p*-value)	−0.206 (.005)	Age

[a] We assume β = −0.001. [b] Cross-lagged regression results (β, *p*-value) not reported for middle-aged adults. [c] The measure of the correlation coefficient was not specified. [d] Study not included in the meta-analysis because compare the same daily step category (i.e., low active: 5000−7499 steps/day).

Abbreviations: ADL, activities of daily living; β, standardized beta coefficient; BDI(–II), Beck Depression Inventory Scale(–version II); BMI, body mass index; CES–D, Center for Epidemiologic Studies–Depression subscale; CI, confidence interval; DASS–21, Depression Anxiety and Stress Scale–21; DS, depressive symptoms; DSM-IV, Diagnostic and Statistical Manual of Mental Disorders–4th edition; GDS–15 or 30, Geriatric Depression Scale–15 or 30; HADS–D, Hospital Anxiety and Depression Scale–Depression subscale; HR, hazard ratio; ICD–9 or 10, International Statistical Classification of Diseases and Related Health Problems–9th or 10th Revision; NA, not applicable; OR, odds ratio; PHQ–9, Patient Health Questionnaire–9; POMS–D, Profile of Mood States–Depression scale; PR, prevalence ratio; PROMIS–D, Patient-Reported Outcomes Measurement Information System–Depression scale; RR, rate ratio; SD, standard deviation; SE, standard error; TQS, Two-Question Screen.

© 2024 Bizzozero-Peroni B et al. *JAMA Network Open*.

eTable 3. List of the studies fully assessed for eligibility and excluded.

**Duplicated data**	
1.	Opoku Asare K, Moshe I, Terhorst Y, Vega J, Hosio S, Baumeister H, et al. Mood ratings and digital biomarkers from smartphone and wearable data differentiates and predicts depression status. *Pervasive Mob Comput*. 2022;83.
**No daily steps-depression association**	
2.	Adachi T, Kono Y, Iwatsu K, Shimizu Y, Yamada S. Duration of moderate to vigorous daily activity is negatively associated with slow walking speed independently from step counts in elderly women aged 75 years or over: A cross-sectional study. *Arch Gerontol Geriatr*. 2018;74:94–9.
3.	Blumenburg WT, Frederick JM, Cross BL, Culver MN, McMillan NK, Montoye AH, et al. Physical fitness, but not physical activity, is associated with mental health in apparently healthy young adults. *J Sports Med Phys Fitness*. 2022;62(10):1410–7.
4.	Coelho CM, Campos LA, Pereira FO, Cardoso RM, Nascimento LM, Oliveira JBL, et al. Objectively measured daily-life physical activity of moderate-to-severe Brazilian asthmatic women in comparison to healthy controls: A cross-sectional study. *J Asthma*. 2018;55(1):73–8.
5.	Dooley EE, Pompeii LA, Palta P, Martinez-Amezcua P, Hornikel B, Evenson KR, et al. Daily and hourly patterns of physical activity and sedentary behavior of older adults: Atherosclerosis risk in communities (ARIC) study. *Prev Med Rep*. 2022;28.
6.	Egerton T, Chastin SFM, Stensvold D, Helbostad JL. Fatigue May Contribute to Reduced Physical Activity Among Older People: An Observational Study. *J Gerontol A Biol Sci Med Sci*. 2016;71(5):670–6.
7.	Eid SW, Brown RF, Maloney SK, Birmingham CL. Can the relationship between overweight/obesity and sleep quality be explained by affect and behaviour? *Eat Weight Disord*. 2022;27(7):2821–34.
8.	Figueroa CA, Vittinghoff E, Aguilera A, Fukuoka Y. Differences in objectively measured daily physical activity patterns related to depressive symptoms in community dwelling women - mPED trial. *Prev Med Rep*. 2021;22.
9.	Furlanetto KC, Mantoani LC, Bisca G, Morita AA, Zabatiero J, Proença M, et al. Reduction of physical activity in daily life and its determinants in smokers without airflow obstruction. *Respirology*. 2014;19(3):369–75.

© 2024 Bizzozero-Peroni B et al. *JAMA Network Open*.

10. Harris TJ, Owen CG, Victor CR, Adams R, Cook DG. What factors are associated with physical activity in older people, assessed objectively by accelerometry? *Br J Sports Med*. 2009;43(6):442–50.
11. Husu P, Tokola K, Vähä-Ypyä H, Sievänen H, Vasankari T. Depressive Symptoms Are Associated With Accelerometer-Measured Physical Activity and Time in Bed Among Working-Aged Men and Women. *J Meas Phys Behav*. 2022;5(3):168–77.
12. Jung S, Lee S, Lee S, Bae S, Imaoka M, Harada K, et al. Relationship between physical activity levels and depressive symptoms in community-dwelling older Japanese adults. *Geriatr Gerontol Int*. 2018;18(3):421–7.
13. Lee PH, Nan H, Yu YY, McDowell I, Leung GM, Lam TH. For non-exercising people, the number of steps walked is more strongly associated with health than time spent walking. *J Sci Med Sport*. 2013;16(3):227–30.
14. Li JW, Guo YT, Di Tanna GL, Neal B, Chen YD, Schutte AE. Vital Signs During the COVID-19 Outbreak: A Retrospective Analysis of 19,960 Participants in Wuhan and Four Nearby Capital Cities in China. *Glob Heart*. 2021;16(1):47.
15. Osugi Y, Imai A, Kurihara T, Kishigami K, Higashida K, Sanada K. Interaction Between Sarcopenic Obesity and Nonlocomotive Physical Activity on the Risk of Depressive Symptoms in Community-Dwelling Older Adult Japanese Women. *J Aging Phys Act*. 2023;1–7.
16. Park C, Atique MMU, Mishra R, Najafi B. Association between Fall History and Gait, Balance, Physical Activity, Depression, Fear of Falling, and Motor Capacity: A 6-Month Follow-Up Study. *Int J Environ Res Public Health*. 2022;19(17).
17. Rykov Y, Thach TQ, Bojic I, Christopoulos G, Car J. Digital Biomarkers for Depression Screening With Wearable Devices: Cross-sectional Study With Machine Learning Modeling. *JMIR Mhealth Uhealth*. 2021;9(10):e24872.
18. Sander C, Ueck P, Mergl R, Gordon G, Hegerl U, Himmerich H. Physical activity in depressed and non-depressed patients with obesity. *Eat Weight Disord*. 2018;23(2):195–203.
19. Staff TE, O'Leary M, Fretts AM. Depression, physical activity, and incident cardiovascular disease among American Indians: The strong heart family study. *Psychiatry Res Commun.* 2023;3(2).
20. Tazawa Y, Liang K ching, Yoshimura M, Kitazawa M, Kaise Y, Takamiya A, et al. Evaluating depression with multimodal wristband-type wearable device: screening and assessing patient severity utilizing machine-learning. *Heliyon*. 2020;6(2):e03274.

© 2024 Bizzozero-Peroni B et al. *JAMA Network Open*.

21. Traczyk J, Dębiec-Bąk A, Skrzek A, Stefańska M. Assessment of the Psychophysical Sphere and Functional Status of Women Aged 75-90 Living Alone and in Nursing Homes. *Int J Environ Res Public Health*. 2021;18(17).

Wrong population

22. Deng CH, Wang JQ, Zhu LM, Liu HW, Guo Y, Peng XH, et al. Association of Web-Based Physical Education with Mental Health of College Students in Wuhan During the COVID-19 Outbreak: Cross-Sectional Survey Study. *J Med Internet Res*. 2020;22(10):e21301.
23. Mishra R, Park C, York MK, Kunik ME, Wung SF, Naik AD, et al. Decrease in Mobility during the COVID-19 Pandemic and Its Association with Increase in Depression among Older Adults: A Longitudinal Remote Mobility Monitoring Using a Wearable Sensor. *Sensors*. 2021;21(9):3090.
24. Pihlaja M, Tuominen PPA, Peräkylä J, Hartikainen KM. Occupational Burnout Is Linked with Inefficient Executive Functioning, Elevated Average Heart Rate, and Decreased Physical Activity in Daily Life - Initial Evidence from Teaching Professionals. *Brain Sciences*. 2022;12(12):1723.
25. Schmidt FM, Mergl R, Minkwitz J, Holdt LM, Teupser D, Hegerl U, et al. Is There an Association or Not?-Investigating the Association of Depressiveness, Physical Activity, Body Composition and Sleep With Mediators of Inflammation. *Front Psychiatry*. 2020;11.
26. Staff TE, O'Leary M, Fretts AM. Depression, physical activity, and incident cardiovascular disease among American Indians: The strong heart family study. *Psychiatry Res Commun*. 2023;3(2):100125.

Wrong exposure

27. Asai Y, Obayashi K, Oume M, Ogura M, Takeuchi K, Yamagami Y, et al. Farming habit, light exposure, physical activity, and depressive symptoms. A cross-sectional study of the HEIJO-KYO cohort. *J Affect Disord*. 2018;241:235–40.
28. Bae S, Jang M, Kim GM, Yang JG, Thapa N, Park HJ, Park H. Nonlinear Associations between Physical Function, Physical Activity, Sleep, and Depressive Symptoms in Older Adults. *J Clin Med*. 2023;12(18):6009.
29. Blodgett J, Mitchell J, Stamatakis E, Chastin S, Hamer M. Associations between the composition of daily time spent in physical activity, sedentary behaviour and sleep and risk of depression: Compositional data analyses of the 1970 British cohort Study. *J Affect Disord*. 2023;320:616-20.
30. Bustamante EE, Wilbur J, Marquez DX, Fogg L, Staffileno BA, Manning A. Physical activity characteristics and depressive symptoms in older Latino adults. *Ment Health Phys Act*. 2013;6(2):69–77.

© 2024 Bizzozero-Peroni B et al. *JAMA Network Open*.

31. Choi KW, Chen CY, Stein MB, Klimentidis YC, Wang MJ, Koenen KC, et al. Assessment of Bidirectional Relationships Between Physical Activity and Depression Among Adults: A 2-Sample Mendelian Randomization Study. *JAMA Psychiatry*. 2019;76(4):399–408.
32. Cohn-Schwartz E, Khalaila R. Accelerometer-Assessed Physical Activity and Cognitive Performance among European Adults Aged 50+: The Mediating Effects of Social Contacts and Depressive Symptoms. *Healthcare (Basel)*. 2022;10(11).
33. Dankel SJ, Loenneke JP, Loprinzi PD. Mild Depressive Symptoms Among Americans in Relation to Physical Activity, Current Overweight/Obesity, and Self-Reported History of Overweight/Obesity. *Int J Behav Med*. 2016;23(5):553–60.
34. de Groot IB, Bussmann JB, Stam HJ, Verhaar JAN. Actual everyday physical activity in patients with end-stage hip or knee osteoarthritis compared with healthy controls. *Osteoarthritis Cartilage*. 2008;16(4):436–42.
35. Difrancesco S, Penninx BWJH, Riese H, Giltay EJ, Lamers F. The role of depressive symptoms and symptom dimensions in actigraphy-assessed sleep, circadian rhythm, and physical activity. *Psychol Med*. 2022;52(13).
36. Dooley EE, Chen L, Ghazi L, Hornikel B, Martinez-Amezcua P, Palta P, Bowling CB, Muntner P, Lewis CE, Pettee Gabriel K. Multimorbidity is associated with lower total 24-hour movement activity among US adults. *Prev Med Rep*. 2023;36:102483.
37. Edler JS, Manz K, Rojas-Perilla N, Baumeister H, Cohrdes C. The role of personality traits and social support in relations of health-related behaviours and depressive symptoms. *BMC Psychiatry*. 2022;22(1).
38. Felez-Nobrega M, Werneck AO, El Fatouhi D, de Luca K, Islam SM, Franzese F. Device-based physical activity and late-life depressive symptoms: An analysis of influential factors using share data. *J Affect Disord*. 2023;322:267-272.
39. Forshaw A, Lee Alfrey K, Maher JP, Rebar AL. But that's who I Am: The inability to enact physical activity identity is associated with depression and anxiety symptoms. *MENPA*. 2023;24.
40. Glaus J, Kang SJ, Guo W, Lamers F, Strippoli MF, Leroux A, Dey D, Plessen KJ, Vaucher J, Vollenweider P, Zipunnikov V, Merikangas KR, Preisig M. Objectively assessed sleep and physical activity in depression subtypes and its mediating role in their association with cardiovascular risk factors. *J Psychiatr Res*. 2023;163:325-336.
41. Iob E, Pingault JB, Munafò MR, Stubbs B, Gilthorpe MS, Maihofer AX; Psychiatric Genomics Consortium Posttraumatic Stress Disorder Working Group; Danese A. Testing the causal relationships of physical activity and sedentary behaviour with mental

© 2024 Bizzozero-Peroni B et al. *JAMA Network Open*.

health and substance use disorders: a Mendelian randomisation study. *Mol Psychiatry*. 2023;28(8):3429-3443.

42. Kawada T, Katsumata M, Suzuki H, Shimizu T. Actigraphic predictors of the depressive state in students with no psychiatric disorders. *J Affect Disord*. 2007;98(1–2):117–20.
43. Konopka MJ, Köhler S, Stehouwer CDA, Schaper NC, Henry RMA, van der Kallen CJH, et al. Accelerometer-derived sedentary time and physical activity and the incidence of depressive symptoms - The Maastricht Study. *Psychol Med*. 2020;52(13):2786–93.
44. Koolhaas CM, Van Rooij FJA, Cepeda M, Tiemeier H, Franco OH, Schoufour JD. Physical activity derived from questionnaires and wrist-worn accelerometers: comparability and the role of demographic, lifestyle, and health factors among a population-based sample of older adults. *Clin Epidemiol*. 2018;10:1.
45. Ku PW, Steptoe A, Liao Y, Sun WJ, Chen LJ. Prospective relationship between objectively measured light physical activity and depressive symptoms in later life. *Int J Geriatr Psychiatry*. 2018;33(1):58–65.
46. Lee SW, Shim JS, Song BM, Lee HJ, Bae HY, Park JH, et al. Comparison of self-reported and accelerometer-assessed measurements of physical activity according to socio-demographic characteristics in Korean adults. *Epidemiol Health*. 2018;40:e2018060.
47. Li YM, Konstabel K, Mõttus R, Lemola S. Temporal associations between objectively measured physical activity and depressive symptoms: An experience sampling study. *Front Psychiatry*. 2022;13:1610.
48. Loprinzi PD. Objectively measured light and moderate-to-vigorous physical activity is associated with lower depression levels among older US adults. *Aging Ment Health*. 2013;17(7):801–5.
49. Loprinzi PD, Cardinal BJ. Interrelationships among physical activity, depression, homocysteine, and metabolic syndrome with special considerations by sex. *Prev Med (Baltim)*. 2012;54(6):388–92.
50. Loprinzi PD, Mahoney S. Concurrent occurrence of multiple positive lifestyle behaviors and depression among adults in the United States. *J Affect Disord*. 2014;165:126–30.
51. Maher JP, Huh J, Intille S, Hedeker D, Dunton GF. Greater variability in daily physical activity is associated with poorer mental health profiles among obese adults. *Ment Health Phys Act*. 2018;14:74–81
52. Nam HK, Park J, Cho SI. Association between depression, anemia and physical activity using isotemporal substitution analysis. *BMC Public Health*. 2023;23(1):2236.

© 2024 Bizzozero-Peroni B et al. *JAMA Network Open*.

53. O'Brien JT, Gallagher P, Stow D, Hammerla N, Ploetz T, Firbank M, et al. A study of wrist-worn activity measurement as a potential real-world biomarker for late-life depression. *Psychol Med*. 2017;47(1):93–102.

54. Osugi Y, Imai A, Kurihara T, Kishigami K, Higashida K, Sanada K. Interaction Between Sarcopenic Obesity and Nonlocomotive Physical Activity on the Risk of Depressive Symptoms in Community-Dwelling Older Adult Japanese Women. *J Aging Phys Act*. 2023;31(4):541-547.

55. Poole L, Steptoe A, Wawrzyniak AJ, Bostock S, Mitchell ES, Hamer M. Associations of objectively measured physical activity with daily mood ratings and psychophysiological stress responses in women. *Psychophysiology*. 2011;48(8):1165–72.

56. Poon CY, Cheng YC, Wong VW, Tam HK, Chung K, Yeung WF, Ho F. Directional associations among real-time activity, sleep, mood, and daytime symptoms in major depressive disorder using actigraphy and ecological momentary assessment. *Behav Res Ther*. 2024;173:104464.

57. Rautio N, Seppänen M, Timonen M, Puhakka S, Kärmeniemi M, Miettunen J, Lankila T, Farrahi V, Niemelä M, Korpelainen R. Associations between neighbourhood characteristics, physical activity and depressive symptoms: the Northern Finland Birth Cohort 1966 Study. *Eur J Public Health*. 2024;34(1):114-120.

58. Rovero M, Preisig M, Marques-Vidal P, Strippoli MF, Vollenweider P, Vaucher J, Berney A, Merikangas KR, Vandeleur CL, Glaus J. Subtypes of major depressive disorders and objectively measured physical activity and sedentary behaviors in the community. *Compr Psychiatry*. 2024;129:152442.

59. Shah RC, Buchman AS, Leurgans S, Boyle PA, Bennett DA. Association of total daily physical activity with disability in community-dwelling older persons: a prospective cohort study. *BMC Geriatr*. 2012;12.

60. Shou H, Cui L, Hickie I, Lameira D, Lamers F, Zhang J, et al. Dysregulation of objectively assessed 24-hour motor activity patterns as a potential marker for bipolar I disorder: results of a community-based family study. *Transl Psychiatry*. 2017;7(8):e1211.

61. Thralls KJ, Godbole S, Manini TM, Johnson E, Natarajan L, Kerr J. A comparison of accelerometry analysis methods for physical activity in older adult women and associations with health outcomes over time. *J Sports Sci*. 2019;37(20):2309–17.

62. Whitaker KM, Sharpe PA, Wilcox S, Hutto BE. Depressive symptoms are associated with dietary intake but not physical activity among overweight and obese women from disadvantaged neighborhoods. *Nutr Res*. 2014;34(4):294–301.

© 2024 Bizzozero-Peroni B et al. *JAMA Network Open*.

63. Zhang Y, Folarin AA, Sun S, Cummins N, Vairavan S, Qian L, et al. Associations Between Depression Symptom Severity and Daily-Life Gait Characteristics Derived From Long-Term Acceleration Signals in Real-World Settings: Retrospective Analysis. *JMIR Mhealth Uhealth*. 2022;10(10).

Wrong outcome

64. Bernard P, Doré I, Romain AJ, Hains-Monfette G, Kingsbury C, Sabiston C. Dose response association of objective physical activity with mental health in a representative national sample of adults: A cross-sectional study. *PLoS One*. 2018;13(10):e0204682.

65. Carels RA, Hlavka R, Selensky JC, Solar C, Rossi J, Caroline Miller J. A daily diary study of internalised weight bias and its psychological, eating and exercise correlates. *Psychol Health*. 2019;34(3):306–20.

66. Chen ST, Yang CH, Hyun J, Ku PW. The association between morning pleasant anticipation and daily positive incidental affect on adults' daily steps: An ecological momentary assessment study. *Psychol Sport Exerc*. 2024;70:102561.

67. Cleland VJ, Schmidt MD, Salmon J, Dwyer T, Venn A. Correlates of pedometer-measured and self-reported physical activity among young Australian adults. *J Sci Med Sport*. 2011;14(6):496–503.

68. Grace JM, Naiker J. The association between objectively measured physical activity and health-related quality of life, life-space mobility and successful ageing in older Indian adults. *Health SA Gesondheid*. 2022;27.

69. Gruenenfelder-Steiger AE, Katana M, Martin AA, Aschwanden D, Koska JL, Kundig Y, et al. Physical activity and depressive mood in the daily life of older adults. *GeroPsych*. 2017;30(3):119–29.

70. LaMonte MJ, LaCroix AZ, Nguyen S, Evenson KR, Di C, Stefanick ML, et al. Accelerometer-Measured Physical Activity, Sedentary Time, and Heart Failure Risk in Women Aged 63 to 99 Years. *JAMA Cardiol*. 2024;9(4):336-345.

Wrong study design

71. Al-Eisa E, Buragadda S, Melam GR. Association between physical activity and psychological status among Saudi female students. *BMC Psychiatry*. 2014;14(1):1–5.

72. Chan LLY, van Schooten KS, Lord SR, Delbaere K, Brodie M. Short Daily-Life Walking Bouts and Poor Self-Reported Health Predict the Onset of Depression in Community-Dwelling Older People: A 2-Year Longitudinal Cohort Study. *J Am Med Dir Assoc*. 2022;23(7):1242-1247.e3.

© 2024 Bizzozero-Peroni B et al. *JAMA Network Open*.

eTable 4. Baseline characteristics of studies included in the systematic review and meta-analysis.

Reference	Country	Study design	n, female (%)	Mean age ± SD or range	BMI (kg/m²)	Step-counting device	Mean steps/day ± SD	Range steps/day[a]	Exposure harmonization (daily steps)	Depression outcome (measure)	DS/DD (%)[b]	Mean DS ± SD
Aaltonen et al.[56] 2023	Finland	Cross-sectional	38 (50)	91.2 ± 1.6	25.2	Accelerometer	2931 ± 2447.6	518–11 190	Increase	DS, continuous	10.5	14.3 ± 6.1
Chan et al.[63] 2023	UK	Prospective cohort (7-y)	72 359 (55.3)	62.0 ± 7.9	26.6	Accelerometer	8071.9 ± 3308.6	NR	Increasing 1000 steps	DE, categorical[c] (ICD–10)	0[d]	NA
Cisek-Wozniak et al.[33] 2021	Poland	Cross-sectional	110 (100)	67.3 ± 6.1	28.1	Pedometer	4462.0 ± 3465.0	746–16 750	Increase, high *vs*. low levels	DS, continuous and categorical[e] (GDS–30)	6.4	3.9 ± 3.3
Daniel et al.[53] 2013	USA	Cross-sectional	110 (62.7)	53.0 ± 5.3	28.6	Accelerometer	6904.3 ± 3388.1	NR	Increase	DS, continuous (CES–D)	50.0	12.5 ± 4.6
Ewald et al.[51] 2014	Australia	Cross-sectional	2458 (52.0)	55–85	28.6	Pedometer	NR	1960–14 930	High *vs*. low levels	DS, continuous (CES–D)	9.3	NR
Fastame et al.[57] 2023	Italy	Cross-sectional	120 (56.7)	82.0 ± 8.4	NR	Accelerometer	NR	NR	Increase	DS, continuous (GDS–15)	NR	NR
Fennell et al.[29] 2022	Spain	Cross-sectional	78 (33.3)	22.4 ± 3.1	NR	Accelerometer	5746.3 ± 5393.2	NR	Increase	DS, continuous (POMS–D)	NR	NR
Fukuie et al.[52] 2023	Japan	Cross-sectional	290 (68.2)	18.6 ± 0.6	20.7	Accelerometer	5946.8 ± 2804.8	834.8–17 941.4	Increase	DS, continuous (BDI–II)	NR	9.3 ± 7.6
Fukukawa et al.[59] 2004	Japan	Panel study (2-y)	314 (41.7)	65–79	NR	Pedometer	5281.0 ± 2214.0	NR	Increase	DS, continuous (CES–D)	NR	6.7 ± 6.9
Hsueh et al.[60] 2021[f]	Taiwan	Prospective cohort (2-y)	274[g] (54.4)	74.5 ± 6.1	NR	Accelerometer	4733.9 ± 3073.6	107–23591	Increasing 1000 steps, high *vs*. low levels	DS, continuous (GDS–15)	17.5	2.2 ± 2.3
Hussenoeder et al.[37] 2022	Germany	Cross-sectional	1451 (52.1)	55.0 ± 11.8	NR	Accelerometer	10 116.3 ± 3660.0	1309–27 878	High *vs*. low levels	DS, continuous (CES–D)	14.3	9.7 ± 6.5
Inada et al.[61] 2021[f]	Japan	Panel study (5-y)	191 (51.8)	69.5 ± 4.1	NR	Accelerometer	NR	NR	High *vs*. low levels	DS, continuous (HADS–D)	NR	4.3 ± 3.4

© 2024 Bizzozero-Peroni B et al. *JAMA Network Open*.

Kangasniemi et al.[50] 2014	Finland	Cross-sectional	108 (79.0)	43.0 ± 5.2	26.0	Accelerometer	9295.5 ± 2973.3	NR	Increase, high *vs.* low levels	DS, continuous (BDI–II)	7.4	5.1 ± 4.4
Langsetmo et al.[49] 2017	USA	Cross-sectional	1283 (0.0)	79.1 ± 5.3	27.1	Accelerometer	5652.0 ± 3092.0	18–18 827	High *vs.* low levels	DS, continuous (GDS–15)	NR	1.7 ± 1.9
Lee et al.[32] 2014	USA	Cross-sectional	810 (54.8)	70.4 ± 9.1	27.9	Accelerometer	7759.0 ± 6316.5	NR	High *vs.* low levels	DS, categorical[h] (PHQ–9)	4.0	NR
Ludwig et al.[55] 2018	UK	Cross-sectional	1720 (14.5)	69.0 ± 4.1	NR	Accelerometer	6389.2 ± 2527.7	105–21 313	Increase	DS, continuous (PHQ–9)	15.7	NR
Maruyama.[48] 2022	Japan	Cross-sectional	52 (100)	70.9 ± 6.0	22.1	Accelerometer	7250.2 ± 2831.3	NR	High *vs.* low levels	DS, continuous (POMS–D)	NR	44.8 ± 5.7
Master et al.[62] 2022	USA	Prospective cohort (4-y)	6042[i] (72.5)	55.2 ± 19.4	28.9	Pedometer	8208.3 ± 3214.9	549–33 600	Increasing 1000 steps, high *vs.* low levels	MDD, categorical[j] (ICD–9)	0[i]	NA
Mckercher et al.[31] 2009	Australia	Cross-sectional	1995[k] (52.4)	31.5 ± 2.6	25.7	Pedometer	NR	NR	High *vs.* low levels	DD, categorical[l] (DSM–IV)	15.5	NA
Moshe et al.[28] 2021	Finland	Cross-sectional	55 (54.5)	42.8 ± 11.6	NR	Accelerometer	10 378.4 ± 1120.3[m]	NR	Increase	DS, continuous (DASS–21)	32.7	3.8 ± 3.5
Proenca et al.[47] 2020	Brazil	Cross-sectional	221 (50.2)	20.6 ± 3.8	22.0	Pedometer	8905.3 ± 2770.2	NR	High *vs.* low levels	DS, continuous (BDI)	NR	5.9 ± 4.1
Raudsepp and Riso.[58] 2017[f]	Estonia	Panel study (2-y)	195 (74.3)	72.1 ± 2.1	NR	Pedometer	6236.2 ± 852.8	NR	Increase	DS, continuous (GDS–15)	0	5.3 ± 2.8
Sato et al.[46] 2021	Japan	Cross-sectional	2846 (59.5)	45.9 ± 11.3	NR	Accelerometer	6590.7 ± 4433.7	NR	High *vs.* low levels	DS, categorical[n] (TQS)	40.0	NR
Shimamoto et al.[45] 2021	Japan	Cross-sectional	85 (38.8)	18.9 ± 1.4	NR	Accelerometer	9513.3 ± 3283.2	4053–21 458	Increase	DS, continuous (PHQ–9)	31.8	5.3 ± 3.2
Spartano et al.[30] 2021	USA	Cross-sectional	1769 (50.8)	47.0 ± 9.0	27.9	Accelerometer	8587.5 ± 3899.1	922 to >10 441	High *vs.* low levels	DS, categorical[o] (CES–D)	9.5	6.2 ± 7.2
Suyama et al.[43] 2015	Japan	Cross-sectional	102 (72.5)	76.5 ± 5.1	22.8	Accelerometer	6848.0 ± 2263.0	NR	Increase	DS, continuous (CES–D)	12.7	9.6 ± 6.1
Tao et al.[44] 2019	China	Cross-sectional	220 (52.2)	20.3 ± 2.4	20.7	Accelerometer	8105.6 ± 2188.9	NR	Increase	DS, continuous (PROMIS–D)	NR	1.8 ± 0.8

© 2024 Bizzozero-Peroni B et al. *JAMA Network Open*.

Thomas and Vejlgaard.[54] 2018	USA	Cross-sectional	12[p] (66.7)	26.1 ± 4.5	NR	Accelerometer	NR	NR	Increase	DS, continuous (BDI–II)	NR	NR
Tonello et al.[42] 2019	Brazil	Cross-sectional	35 (100)	34.5 ± 5.1	26.0	Accelerometer	8597.0 ± 3204.0	3257–15 464	Increase	DS, continuous (BDI)	62.8	12.3 ± 7.1
Vallance et al.[41] 2013	Canada	Cross-sectional	385 (0.0)	65.3 ± 7.5	26.9	Pedometer	8539.0 ± 4244.0	<3103 to 22 316	High *vs*. low levels	DS, continuous (CES–D)	NR	7.8 ± 7.8
Varma et al.[40] 2014	USA	Cross-sectional	187 (76.5)	66.8 ± 5.6	NR	Accelerometer	6888.1 ± (2835.9)	NR	Increasing 1000 steps	DS, continuous (GDS–15)	NR	3.1 ± 1.5
Ward-Ritacco et al.[39] 2014	USA	Cross-sectional	74[q] (100)	58.9 ± 3.8	25.6	Accelerometer	8814.0 ± 3352.0	2632–17 393	Increase	DS, continuous (BDI–II)	15.0	6.9 ± 6.8
Yoshiuchi et al.[38] 2006	Japan	Cross-sectional	184 (54.8)	65–85	NR	Accelerometer	6635.0 ± 2750.0	953–13 670	Increase	DS, continuous (HADS–D)	4.3	3.4 ± 2.6

[a] Minimum (or lowest category) and maximum (or highest category) of steps/day. [b] Percentage of diagnosis of depression or mild-to-severe depressive symptoms at baseline according to each assessment measure used in the included studies. [c] Defined as the first occurrence of a depressive episode. [d] The incidence of depression was 1.8%. [e] Defined as mild-to-severe depressive symptoms according to the scale and cut-off point (≥10) used. [f] Cross-sectional data were also included in the meta-analysis. [g] Participants with available data in the prospective analysis, n=226. [h] Defined as moderate to severe depressive symptoms according to the scale cut-off point (≥10) used. [i] The study sample in the prospective analyses were n=4884 (Cox regression model) and n=5370 (logistic regression model) with an incidence of depression of 9.0%. [j] Defined as diagnosis of major depressive disorder. [k] Participants with available data in the final analysis, n=1635. [l] Defined as diagnosis of major or persistent depressive disorder. [m] The data were presented in Asare et al. 2022 (doi: 10.1016/j.pmcj.2022.101621). [n] Defined as presence of depressive symptoms according to the scale and cut-off point (≥1) used. [o] Defined as presence of depressive symptoms according to the scale and cut-off point (≥16) used. [p] Participants with available data at the final analysis, n=11. [q] Participants with available data at the final analysis, n=70.

Abbreviations: BDI(–II), Beck Depression Inventory Scale(–version II); BMI, body mass index; CES–D, Center for Epidemiologic Studies–Depression subscale; DASS–21, Depression Anxiety and Stress Scale–21; DD, depressive disorder; DE, depressive episode; DS, depressive symptoms; DSM-IV, Diagnostic and Statistical Manual of Mental Disorders–4th edition; GDS–15 or 30, Geriatric Depression Scale–15 or 30; HADS–D, Hospital Anxiety and Depression Scale–Depression subscale; ICD–9 or 10, International Statistical Classification of Diseases and Related Health Problems–9th or 10th Revision; MDD, major depressive disorder; NA, not applicable; NR, not reported; PHQ–9, Patient Health Questionnaire–9; POMS–D, Profile of Mood States–Depression scale; PROMIS–D, Patient-Reported Outcomes Measurement Information System–Depression scale; SD, standard deviation; TQS, Two-Question Screen; UK, United Kingdom; USA, United States of America.

© 2024 Bizzozero-Peroni B et al. *JAMA Network Open*.

eTable 5. Characteristics of the populations, exposures, and outcomes of the included studies.

Reference	Type of population[a]	Device and wearing protocol			Valid step count data		Depression assessment		
		Type (model, brand, acceleration axis)	Days worn	Worn on	Minimum hours/day	Days required	Measure (items, score range)	Evaluation time	Cut-off score of DS[b]
Aaltonen et al.[56] 2023	Nonagenarian twins who could engage in physical activity	Accelerometer (UKK RM42, UKK Institute, triaxial)	7	Hip	10	≥4	CES-D (20, 0–60)	Previous week	>20
Chan et al.[63] 2023	No history of depression	Accelerometer (AX3, Axivity, triaxial)	7	Wrist	NR	NR	ICD–10 (diagnosis)	First occurrence[c]	NA
Cisek-Wozniak et al.[33] 2021	No medical contraindications	Pedometer (Forerunner 35 HR, Garmin, triaxial)	7	Wrist	NR	NR	GDS (30, 0–30)	Previous week	>10
Daniel et al.[53] 2013	South Asian Indian immigrants without disability	Accelerometer (Lifecorder EX NL2200, Suzuken, uniaxial)	7	Waist	NR	≥3	CES-D (12, 0–48)	Previous week	>10
Ewald et al.[51] 2014	-	Pedometer (DW200, Yamax, uniaxial)	7	Waist	≥9	≥3	CES-D (20, 0–60)	Previous week	NR
Fastame et al.[57] 2023	Community dwellers without cognitive, musculoskeletal, or neurologic condition	Accelerometer (GT3X, ActiGraph, triaxial)	7	Wrist	≥16	NR	GDS (15, 0–15)	Previous week	>10
Fennell et al.[29] 2022	University students	Accelerometer (Mi Band 2, Xiaomi, triaxial)	7	Wrist	≥10	NR	POMS (15, 0–60)	Previous week	NR
Fukuie et al.[52] 2023	Community dwellers without disability	Accelerometer (MC-500, Yamasa, uniaxial)	7	Waist	NR	5	CES-D (20, 0–60)	Previous week	NR
Fukukawa et al.[59] 2004	First-year university students	Pedometer (Select II, Suzuken, uniaxial)	7	NR	NR	NR	BDI–II (21, 0–63)	Previous 2-week	NR
Hsueh et al.[60] 2021	Community dwellers walking independently	Accelerometer (GT3X+, ActiGraph, triaxial)	7	Waist	≥10	≥4 + ≥1[d]	GDS (15, 0–15)	Previous week	≥5
Hussenoeder et al.[37] 2022	-	Accelerometer (SenseWear Pro 3, BodyMedia, triaxial)	7	Arm	≥20	≥4 + ≥1[d]	CES-D (20, 0–60)	Previous week	≥16

© 2024 Bizzozero-Peroni B et al. *JAMA Network Open*.

Inada et al.[61] 2021	No walking disability	Accelerometer (Lifecorder, Suzuken, uniaxial)	31	Waist	NR	NR	HADS-D (7, 0–21)	Previous 2-week	NR
Kangasniemi et al.[50] 2014	-	Accelerometer (GT1M, ActiGraph, biaxial)	7	Hip	≥8.3	≥3	BDI–II (21, 0–63)	Previous 2-week	≥14
Langsetmo et al.[49] 2017	Community dwellers walking independently	Accelerometer (SenseWear Pro 3, BodyMedia, triaxial)	7	Arm	NR[e]	≥5	GDS (15, 0–15)	Previous week	NR
Lee et al.[32] 2014	Community dwellers	Accelerometer (ActiGraph AM-7164, uniaxial)	7	NR	≥10	≥1	PHQ (9, 0–27)	Previous 2-week	≥10
Ludwig et al.[55] 2018	No diagnosed disease, ≥20% risk of cardiovascular events	Accelerometer (GT3X, ActiGraph, triaxial)	7	Waist	≥9	≥5	PHQ (9, 0–27)	Previous 2-week	≥5
Maruyama.[48] 2022	Community dwellers	Accelerometer (Lifecorder GS, Suzuken, uniaxial)	~90	NR	NR[f]	NR	POMS (NR)	Previous week	NR
Master et al.[62] 2022	-	Pedometer (Fitbit devices, triaxial)	≥168	Wrist	≥10 [g]	≥15	ICD–9 (diagnosis)	Previous 12 months	NA
Mckercher et al.[31] 2009	-	Pedometer (Digiwalker SW-200, Yamax, uniaxial)	7	NR	>8	≥4	DSM-IV (diagnosis)	Previous 12 months	NA
Moshe et al.[28] 2021	-	Accelerometer (Oura Ring, Oura Health, triaxial)	30	Finger (hand)	NR	NR	DASS (21, 0–21)	Previous week	≥5
Proenca et al.[47] 2020	University students	Pedometer (Digiwalker SW-200, Yamax, uniaxial)	7	Waist	≥10	NR	BDI (21, 0–63)	Previous 2-week	≥11
Raudsepp and Riso.[58] 2017	Community dwellers with no medical contraindications	Pedometer (Digiwalker SW-200-024, Yamax, uniaxial)	7	Waist	NR[h]	NR	GDS (15, 0–15)	Previous week	>6
Sato et al.[46] 2021	-	Accelerometer (user's mobile device, triaxial)	124	NR	NR	NR	TQS (2, 0–2)	Previous 4-week	≥1
Shimamoto et al.[45] 2021	First-year university students	Accelerometer (Lifecorder, Suzuken, uniaxial)	14	Waist	NR	NR	PHQ (9, 0–27)	Previous week	≥5

© 2024 Bizzozero-Peroni B et al. *JAMA Network Open*.

Spartano et al.[30] 2021	-	Accelerometer (Actical 198-0200-00, Phillips Respironics, biaxial)	8	Hip	>10[i]	≥3	CES-D (20, 0–60)	Previous week	≥16
Suyama et al.[43] 2015	No diagnosed disease	Accelerometer (Lifecorder GS, Suzuken, uniaxial)	15	Waist	>12	NR	CES-D (20, 0–60)	Previous week	≥16
Tao et al.[44] 2019	University students	Accelerometer (GT9X, ActiGraph, triaxial)	7	Wrist	≥12	≥2 + ≥1[d]	PROMIS–D (4, 0–20)	Previous week	NR
Thomas and Vejlgaard.[54] 2018	No disability	Accelerometer (Misfit Flash, Misfit, triaxial)	14	Wrist	NR	NR	BDI (21, 0–63)	Previous 2-week	NR
Tonello et al.[42] 2019	University employees without diagnosed disease	Accelerometer (GT1M, ActiGraph, biaxial)	7	Hip	NR	5[j]	BDI (21, 0–63)	Previous 2-week	≥10
Vallance et al.[41] 2013	-	Pedometer (SC-01, StepsCount, uniaxial)	3	NR	NR	NR	CES-D (20, 0–60)	Previous week	NR
Varma et al.[40] 2014	Without cognitive impairment	Accelerometer (StepWatch, triaxial)	3–7	Ankle	≥6[k]	NR	GDS (15, 0–15)	Previous week	NR
Ward-Ritacco et al.[39] 2014	Postmenopausal women without diagnosed disease	Accelerometer (1000, New Lifestyles, uniaxial)	7	Hip	≥10	≥4	BDI (21, 0–63)	Previous 2-week	NR
Yoshiuchi et al.[38] 2006	No diagnosed disease, rural area	Accelerometer (Kenz Lifecorder, Suzuken, uniaxial)	365	Waist	NR	NR	HADS-D (7, 0–21)	Previous 2-week	≥11

[a] Population selection based on specific criteria other than age or sex (studies without specific criteria were left blank with a dash). [b] Cut-off point for determining mild-to-severe depressive symptoms according to each scale used. [c] After the collection of accelerometry data. [d] Weekdays + weekend. [e] ≥90% wear time. [f] ≥500 steps. [g] ≥100 steps. [h] ≥1000 steps. [i] Light-intensity physical activity time were considered only between 6 AM and 10 PM. [j] Consecutive days. [k] ≥201 steps.

Abbreviations: BDI, Beck Depression Inventory Scale; CES–D, Center for Epidemiologic Studies–Depression subscale; DASS, Depression Anxiety and Stress Scale; DS, depressive symptoms; DSM-IV, Diagnostic and Statistical Manual of Mental Disorders–4th edition; GDS, Geriatric Depression Scale; HADS–D, Hospital Anxiety and Depression Scale–Depression subscale; ICD–9, International Statistical Classification of Diseases and Related Health Problems–9th Revision; NA, not applicable; NR, not reported; PHQ, Patient Health Questionnaire; POMS–D, Profile of Mood States–Depression scale; PROMIS–D, Patient-Reported Outcomes Measurement Information System–Depression scale; TQS, Two-Question Screen.

© 2024 Bizzozero-Peroni B et al. *JAMA Network Open*.

eTable 6. Quality assessment of included studies.[a]

Reference	1	2	3	4	5	6	7	8	9	10	11	12	13	14	Score	Quality
**Cross-Sectional**																
Aaltonen et al.[56] 2023	Y	Y	N	Y	N	N	N	N	Y	N	Y	N	NA	N	5	Fair
Cisek-Wozniak et al.[33] 2021	Y	Y	CD	Y	N	N	N	Y	N	N	Y	N	NA	N	5	Fair
Daniel et al.[53] 2013	Y	Y	Y	Y	Y	N	N	N	Y	N	Y	N	NA	N	7	Fair
Ewald et al.[51] 2014	Y	Y	N	Y	Y	N	N	Y	Y	N	Y	N	NA	Y	8	Good
Fastame et al.[57] 2023	Y	Y	Y	Y	N	N	N	N	Y	N	Y	N	NA	N	6	Fair
Fennell et al.[29] 2022	Y	Y	N	Y	N	N	N	N	Y	Y	Y	N	NA	Y	7	Fair
Fukuie et al.[52] 2023	Y	Y	Y	Y	N	N	N	N	N	N	Y	N	NA	N	5	Fair
Hussenoeder et al.[37] 2022	Y	Y	N	Y	Y	N	N	Y	Y	N	Y	N	NA	Y	8	Good
Kangasniemi et al.[50] 2014	Y	Y	CD	Y	N	N	N	Y	Y	N	Y	N	NA	N	6	Fair
Langsetmo et al.[49] 2017	Y	Y	N	Y	N	N	N	Y	Y	N	Y	N	NA	N	6	Fair
Lee et al.[32] 2014	Y	Y	N	Y	Y	N	N	Y	Y	N	Y	N	NA	Y	8	Good
Ludwig et al.[55] 2018	Y	Y	Y	Y	Y	N	N	N	Y	N	Y	N	NA	N	7	Fair
Maruyama.[48] 2022	Y	Y	CD	Y	N	N	N	Y	N	N	Y	N	NA	N	5	Fair
Mckercher et al.[31] 2009	Y	Y	N	Y	Y	N	N	Y	Y	N	Y	NR	NA	Y	8	Good
Moshe et al.[28] 2021	Y	Y	CD	Y	Y	N	N	N	N	N	Y	N	NA	N	5	Fair
Proenca et al.[47] 2020	Y	Y	Y	Y	Y	N	N	Y	Y	N	Y	N	NA	N	8	Good
Sato et al.[46] 2021	Y	Y	Y	Y	N	N	N	Y	N	N	Y	N	NA	Y	7	Fair
Shimamoto et al.[45] 2021	Y	Y	Y	Y	N	N	N	N	N	N	Y	N	NA	N	5	Fair
Spartano et al.[30] 2021	Y	Y	Y	Y	Y	N	N	Y	Y	N	Y	N	NA	N	8	Good
Suyama et al.[43] 2015	Y	Y	Y	Y	N	N	N	N	Y	N	Y	N	NA	N	6	Fair
Tao et al.[44] 2019	Y	Y	CD	Y	N	N	N	N	Y	N	Y	N	NA	N	5	Fair
Thomas and Vejlgaard.[54] 2018	Y	Y	CD	Y	N	N	N	N	N	N	Y	N	NA	N	4	Poor
Tonello et al.[42] 2019	Y	Y	CD	Y	N	N	N	N	Y	N	Y	N	NA	N	5	Fair
Vallance et al.[41] 2013	Y	Y	Y	Y	N	N	N	Y	N	N	Y	N	NA	Y	7	Fair
Varma et al.[40] 2014	Y	Y	N	Y	N	N	N	N	Y	N	Y	N	NA	Y	6	Fair
Ward-Ritacco et al.[39] 2014	Y	Y	N	Y	N	N	N	N	Y	N	Y	N	NA	N	5	Fair
Yoshiuchi et al.[38] 2006	Y	Y	CD	Y	N	N	N	N	N	N	Y	N	NA	N	4	Poor
**Longitudinal**																
Chan et al.[63] 2023	Y	Y	N	Y	Y	Y	Y	N	N	N	Y	Y	Y	Y	9	Fair
Fukukawa et al.[59] 2004	Y	N	N	Y	N	Y	Y	N	Y	Y	Y	N	Y	Y	9	Fair
Hsueh et al.[60] 2021	Y	Y	Y	Y	N	Y	Y	Y	Y	N	Y	N	Y	Y	11	Good

© 2024 Bizzozero-Peroni B et al. *JAMA Network Open*.

Inada et al.[61] 2021	Y	N	CD	Y	N	Y	Y	Y	N	Y	Y	N	Y	N	8	Fair
Master et al.[62] 2022	Y	Y	N	Y	Y	Y	Y	Y	Y	Y	Y	Y	Y	Y	13	Good
Raudsepp and Riso.[58] 2017	Y	Y	CD	Y	N	Y	Y	N	N	Y	Y	N	Y	Y	9	Fair

[a] Numbers represent the questions included in the National Institutes of Health's Quality Assessment Tool for Observational Cohort and Cross-Sectional Studies: 1. Question clear? 2. Population clearly defined? 3. >50% participants o non-convenience sample? 4. Recruitment populations consistent? 5. Sample size justified? 6. Exposure assessed prior to outcome? 7. Sufficient timeframe? 8. Different exposure levels? 9. Valid exposure? 10. Repeated exposure assessment? 11. Valid outcomes? 12. Outcome assessors blinded? 13. Loss to follow up <20%? 14. Confounders adjusted for? Items 6 and 7 are limitations inherent to cross-sectional study designs. Item 13 was considered not applicable for cross sectional studies. Each cross-sectional and longitudinal study was rated as good, fair, or poor according to the National Institutes of Health's Quality Assessment Tool for Observational Cohort and Cross-Sectional quality rating guide.
Most of the cross-sectional studies (77.8%) had methodological limitations in six key criteria: (i) the proportion of eligible individuals; (ii) the rationale for the sample size; (iii) the use of different levels of exposure; (iv) the repeated assessment of exposure levels; (v) the blinding of the outcome assessors to the exposure status of the participants; and (vi) the inclusion of potential confounding variables in adjusted statistical analyses. Similarly, most longitudinal studies (66.7%) lacked information on four key criteria: (i) the proportion of eligible individuals; (ii) the rationale for sample size; (iii) a clearly defined set of exposure measures; and (iv) the blinding of the outcome assessors to the participants' exposure status.
Abbreviations: CD, cannot determine; N, no; NA, not applicable; NR, not reported; Y, yes.

© 2024 Bizzozero-Peroni B et al. *JAMA Network Open*.

eTable 7. Daily steps and risk of depression in prospective cohort studies.

Studies	Follow-up, mean range (y)	n	Incident cases	RR (95% CI)	*P* value	I^2 (%)	*P* value
Increasing 1000 steps/day[a]	3.9–7.4	77 729	1815[a]	0.91 (0.87–0.94)	<.001	48.3	.16
High (≥7000) *vs*. low (<7000) levels of daily steps[b]	1.8–3.9	5110	515[b]	0.69 (0.62–0.77)	<.001	0	.62

[a] Incident cases of depressive episodes or major depressive disorders. The studies included in this meta-analysis were those conducted by Chan et al. 2023[63] (HR=0.88; 95% CI:0.82–0.93) and Master et al. 2022[62] (OR=0.92, 95% CI:0.89–0.95), [b] Incident cases of major depressive disorders or mild-to-severe depressive symptoms. The studies included in this meta-analysis were those conducted by Hsueh et al. 2021[60] (RR=0.66; 95% CI:0.49–0.89) and Master et al. 2022[62] (HR=0.70, 95% CI:0.59–0.84),
Abbreviations: CI, confidence interval; NA, not applicable; NR, not reported; RR, risk ratio.

© 2024 Bizzozero-Peroni B et al. *JAMA Network Open*.

eTable 8. Subgroup analyses for cross-sectional associations between daily steps and depressive symptoms.

Meta-analysis of correlations[a]	n	Estimate (95% CI)	*P* value	*P* value subgroup	I^2 (%)	*P* value
**Adult age range[b]**						
18–35y	6	0.01 (−0.21 to 0.23)	.91	.23	72.1	.003
36–64y	4	−0.21 (−0.32 to −0.09)	<.001		0	.56
≥65y	9	−0.16 (−0.23 to −0.09)	<.001		32.1	.16
**Step counter type**						
Accelerometer	17	−0.10 (−0.18 to −0.01)	.03	.07	63.5	<.001
Pedometer	2	−0.26 (−0.40 to −0.11)	.001		41.0	.19
**Step counter position[c]**						
Hip	4	−0.11 (−0.26 to 0.05)	.18	.78	12.6	.33
Waist	8	−0.09 (−0.24 to 0.06)	.26		83.4	<.001
Wrist	5	−0.16 (−0.29 to −0.02)	.02		26.4	.25
**Acceleration axis**						
Uniaxial	7	−0.07 (−0.24 to 0.11)	.44	.79	85.0	<.001
Biaxial	2	−0.14 (−0.40 to 0.15)	.36		58.0	.12
Triaxial	10	−0.14 (−0.21 to −0.06)	<.001		0	.56
**Meta-analysis of SMDs[d]**						
**Adult age range[b]**						
18–64y[e]	5	−0.32 (−0.61 to −0.03)	.03	.75	81.8	<.001
≥65y	7	−0.27 (−0.41 to −0.13)	<.001		28.9	.21
**Sex**						
Female	4	−0.29 (−0.46 to −0.11)	.001	.62	71.0	.02
Male	4	−0.23 (−0.40 to −0.06)	.01		45.3	.14
**Step counter type**						
Accelerometer	7	−0.36 (−0.60 to −0.12)	.003	.34	76.4	<.001
Pedometer	5	−0.24 (−0.33 to −0.14)	<.001		16.5	.31
**Step counter position[f]**						
Arm	2	−0.23 (−0.57 to 0.10)	.17	.53	75.1	.05
Hip	2	−0.57 (−1.31 to 0.16)	.13		91.7	<.001
Waist	3	−0.17 (−0.23 to −0.11)	<.001		0	.97

© 2024 Bizzozero-Peroni B et al. *JAMA Network Open*.

**Acceleration axis**						
Uniaxial	7	−0.24 (−0.34 to −0.13)	<.001	.67	4.3	.39
Biaxial	2	−0.57 (−1.31 to 0.16)	.13		91.7	<.001
Triaxial	3	−0.27 (−0.53 to −0.01)	.04		66.6	.05
**Methodological quality**						
Good	6	−0.20 (−0.33 to −0.07)	.002	.12	41.7	.13
Fair	6	−0.42 (−0.65 to −0.18)	<.001		68.1	.008

[a] Since none of the studies stratified results by sex and only one high-quality study was included, subgroup analyses based on these characteristics were not performed. [b] The studies were classified as adults aged 18–35y, 36–64y or ≥65y according to the mean age reported. [c] Other step counter positions (i.e., ankle or toe) not repeated in more than one study were not included in the subgroup analysis. [d] Pooled standardized mean differences between high (≥7500) *vs*. low (<7500) levels of daily steps and depression as a diagnosis or symptoms. [e] Adults aged 18–64y were grouped together due to the small number of studies conducted in young adults. [f] Other step counter positions (i.e., wrist) not repeated in more than one study were not included in the subgroup analysis.
n represents the number of studies included in each subgroup analysis.
Abbreviations: CI, confidence interval; SMD, standardized mean difference.

© 2024 Bizzozero-Peroni B et al. *JAMA Network Open*.

eTable 9. Meta-regression analyses for cross-sectional associations between daily steps and depression.[a]

Covariates	n	Coefficients			Heterogeneity			Test of residual heterogeneity		
		Estimate	SE	*P* value	I^2 (%)	H^2	R^2 (%)	Q	df	*P* value
**Meta-analysis of correlations[b]**										
Age (mean years)	19	−0.003	0.002	.10	76.9	4.3	9.4	37.5	17	.003
Sex (% female)	19	−0.001	0.002	.68	75.6	4.1	0	51.8	17	<.001
**Meta-analysis of SMDs[c]**										
Age (mean years)	12	−0.000	0.004	.99	87.2	7.8	0	31.7	10	<.001
Sex (% female)	12	−0.002	0.003	.58	87.7	8.1	0	30.8	10	<.001
BMI (mean kg/m^2)	10	−0.005	0.040	.87	78.8	4.7	0	20.1	8	.01

[a] Univariate random-effects meta-regression models were used to estimate the proportion of between-study heterogeneity explained by each covariate (for pooled correlation coefficients: mean age [ranging from 18.6–76.5 years] and percentage of females [ranging from 14.5–100%]; for pooled SMDs: mean age [ranging from 20.6–79.1 years], percentage of females [ranging from 0–100%], and body mass index [ranging from 22.0–28.6 kg/m^2]), as well as the change in the effect size estimates for each 1-unit change in the characteristic included as a predictor in the model. For example, each 1-year increment was associated with a reduction in the pooled correlation coefficient estimate (r=−0.003) of the associations between the number of daily steps and depressive symptoms. The estimates from the meta-regression analyses are shown in **eFigure 1**. The variability explained by each model was tested using the Wald test. [b] Pooled correlation coefficients between higher number of daily steps and depressive symptoms. Due to the limited number of studies (n<10) that reported the mean body mass index (n=8), we were unable to perform meta-regressions to examine whether this covariate influenced heterogeneity. [c] Pooled standardized mean differences between high (≥7500) *vs*. low (<7500) levels of daily steps and depression as a diagnosis or symptoms.
n represents the number of studies included in each meta-regression model.
Abbreviations: BMI, body mass index; SE, standard error; SMD, standardized mean difference.

© 2024 Bizzozero-Peroni B et al. *JAMA Network Open*.

eTable 10. Sensitivity analyses excluding studies with data during the COVID-19 lockdown and for depression as categorical outcome.

Analysis	n	Estimate (95% CI)	*P* value	I^2 (%)	*P* value
Excluding data analyzed during the COVID-19 lockdown[a]	17	*r*=−0.11 (−0.20 to −0.02)	.02	68.8	<.001
Depression as categorical outcome[b]	4	OR=0.58 (0.39−0.86)	.006	0	.47

[a] Pooled cross-sectional correlation coefficient between higher number of daily steps and depressive symptoms. The studies conducted by Fennell et al. 2022[29] and Moshe et al. 2021[28] were excluded. [b] Pooled cross-sectional odds ratios between high (≥7500) *vs*. low (<7500) levels of daily steps and depression. The studies included in this meta-analysis were those conducted by Cizek-Wozniak et al. 2021[33] (OR=0.33; 95% CI:0.02–6.05), Lee et al. 2014[32] (OR=0.28, 95% CI:0.09–0.91), McKercher et al. 2009[31] (OR=0.58; 95% CI:0.42–0.81), and Spartano et al. 2022[30] (OR=0.69; 95% CI:0.50–0.95), with a total of 4370 adults and 357 prevalent cases of major depressive disorders or mild-to-severe depressive symptoms.
n represents the number of studies included in each sensitivity analysis.
Abbreviations: CI, confidence interval; OR, odds ratio.

© 2024 Bizzozero-Peroni B et al. *JAMA Network Open*.

eTable 11. Meta-bias for the cross-sectional associations between daily steps and depressive symptoms.

Exposure harmonization (ES)	n	Intercept	Estimate (SE)	*P* value
Continuous (*r*)[a]	19	−0.156	0.389 (0.734)	.60
Categorical (SMD)[b]	12	−0.064	−2.046 (0.545)	.004

[a] Pooled correlation coefficients between higher number of daily steps and depressive symptoms. [b] Pooled standardized mean differences between high (≥7500) *vs*. low (<7500) levels of daily steps and depression as a diagnosis or symptoms.
n represents number of studies included in each analysis.
Abbreviations: ES, effect size; SE, standard error; SMD, standardized mean difference.

© 2024 Bizzozero-Peroni B et al. *JAMA Network Open*.

eTable 12. Recommendations for future studies on the associations between objectively measured daily steps and depression in the general adult population.

Study characteristics	Recommendations
Population	Considering gender differences in depression, in addition to age-related physiological and social changes for both physical activity and mental health, it is essential to conduct studies, specifically prospective cohorts, in both sexes and in adults of all ages with data disaggregated by these characteristics. Furthermore, specific daily step requirements may differ according to depression phenotype (e.g., depressive disorders, profile of depressive symptoms) and associated factors such as current job, family history of depression, genetic variants, social support, or socioeconomic status.
Exposure (daily steps)	Future studies on this topic should monitor daily steps at repeated assessments or continuously throughout a suitable follow-up period to improve the understanding of an individual's physical activity pattern. This approach would be useful not only minimize over or underestimation of the accuracy of step counts and potential biases (e.g., short monitoring periods are prone to an observer effect) but also to assess daily steps through a more realistic and in-depth approach. Indeed, the background level of daily physical activity is likely to vary over time due to several influencing factors, such as season, available leisure time, and work-related stress. In addition to a model that analyzes changes in daily steps over an individual's entire follow-up period rather than a short snapshot, the inclusion of other methods of physical activity assessment (e.g., heart rate monitoring) could reduce bias and inaccuracies. Furthermore, the accuracy of step counter devices may fluctuate not only in terms of the device type but also in terms of the manufacturer, model and acceleration axes, or the shape and position on the body on which they are positioned. A more detailed examination of these exposure factors (e.g., studies using more than one type of device or step counter position) and the disclosure of specific criteria for valid step count assessment will increase the precision of studies estimating daily physical activity levels. It is recommended that future studies provide comprehensive details regarding the devices utilized, the protocols employed for their application, and the validity of the step count data obtained. In addition, a more comprehensive analytical approach that goes beyond step count could facilitate a more nuanced understanding of the relationship between daily steps and depression. By examining patterns, variability, and the intensity of daily steps, future studies may be able to identify more sensitive indicators of mental health status. These complementary metrics (e.g., intraindividual variance, circadian rhythm of steps, and entropy of steps) could help design more tailored public health strategies for depression prevention that focus not only on increasing physical activity but also on optimizing the rhythm, intensity, and regularity of movement throughout the day. Finally, to facilitate interpretation and comparison across various studies, it is advisable to analyze daily steps both as a continuous and categorical variable. We propose employing categories of daily steps related to the estimated levels of habitual physical activity in adults, as indicated by prior evidence. Furthermore, as a minimum of 5000 steps/day could signify a high number of steps for specific populations (e.g., older adults), it is recommended to consider other cut-off points that disaggregate the potentially sedentary lifestyle category (<5000 steps/day).
Outcome (depression)	Future observational studies should consider depression outcome as a categorical variable, distinguishing between individuals without a clinical diagnosis; those diagnosed with a depressive disorder; and considering the severity of depressive symptoms, encompassing mild, moderate, and/or severe manifestations. Furthermore, there is a need for research that explores the associations of daily steps with depressive symptoms,

© 2024 Bizzozero-Peroni B et al. *JAMA Network Open*.

	considering their specific attributes such as mood and somatic components. Moreover, the assessment of depression using self-report scales could lead to inconsistencies and some degree of measurement error due to information bias. Further studies are needed to provide results based on the clinical diagnosis of depression.
Study design	Given the potential for reverse causality between physical activity and depression, large and long-term prospective cohort studies are sorely needed. These studies should include representative data from the general adult population who were free of depression at the baseline assessment and analyze the risk of depression or changes in depressive symptoms over an appropriate follow-up period. This is especially important in studies on the incidence of mental disorders, where sufficient time is required to observe changes in depressive symptom patterns. Our results suggest that further high-quality cohort studies examining the association of real-world unstructured physical activity, as indicated by daily step counts, and its potential role in the prevention of depression are needed. Such studies could provide crucial evidence for an analytical approach that includes complementary metrics beyond step count, such as intraindividual variance or the entropy of steps. This would allow the identification of specific needs according to the depression phenotype and associated factors such as age or sex. In addition, future community trials are needed to assess whether and to what extent the promotion of increased daily steps in population settings may be an effective strategy for the prevention of depression. In terms of the methodological quality of cross-sectional and longitudinal studies, improvements are required in the following criteria: (i) the proportion of eligible individuals; (ii) the rationale for the sample size; (iii) the use of different levels of exposure; (iv) a clearly defined set of exposure measures; (v) the repeated assessment of exposure levels; (vi) the blinding of the outcome assessors to the exposure status of the participants; and (vii) the inclusion of potential confounding variables in adjusted statistical analyses. Finally, it is recommended that future reviews consider individual patient data meta-analyses, which would allow for more detailed assessments of participant-level moderators.
Other	Future observational studies should consider mediation/moderation analyses and appropriate covariate adjustments according to previous relevant evidence on the daily steps-depression relationship, such as alcohol use, age, body mass index, cohabitation status, household economy, race, sex, smoking status, social activities, type of employment, or wear time. Moreover, other potential confounding factors that have rarely been considered and could be critical for the study association could be characteristics (e.g., frequency, time) of physical activity modalities without steps such as swimming or rowing, dietary patterns, family history of mental disorders, loneliness, physical fitness, season, sleep quality, step intensity (e.g., bout cadence steps per minute), or walkability of urban-rural environments. To gain deeper insight into the relationship between daily steps and depression prevention in the general adult population, further research is needed to elucidate the mechanisms by which daily steps influence depression. Furthermore, a better understanding of this phenomenon can be achieved by investigating the specific circumstances in which daily steps affect depressive symptoms and those in which they do not. Finally, it is recommended that future reviews consider individual patient meta-analyses, which would facilitate more detailed assessments of participant-level moderators.

© 2024 Bizzozero-Peroni B et al. *JAMA Network Open*.

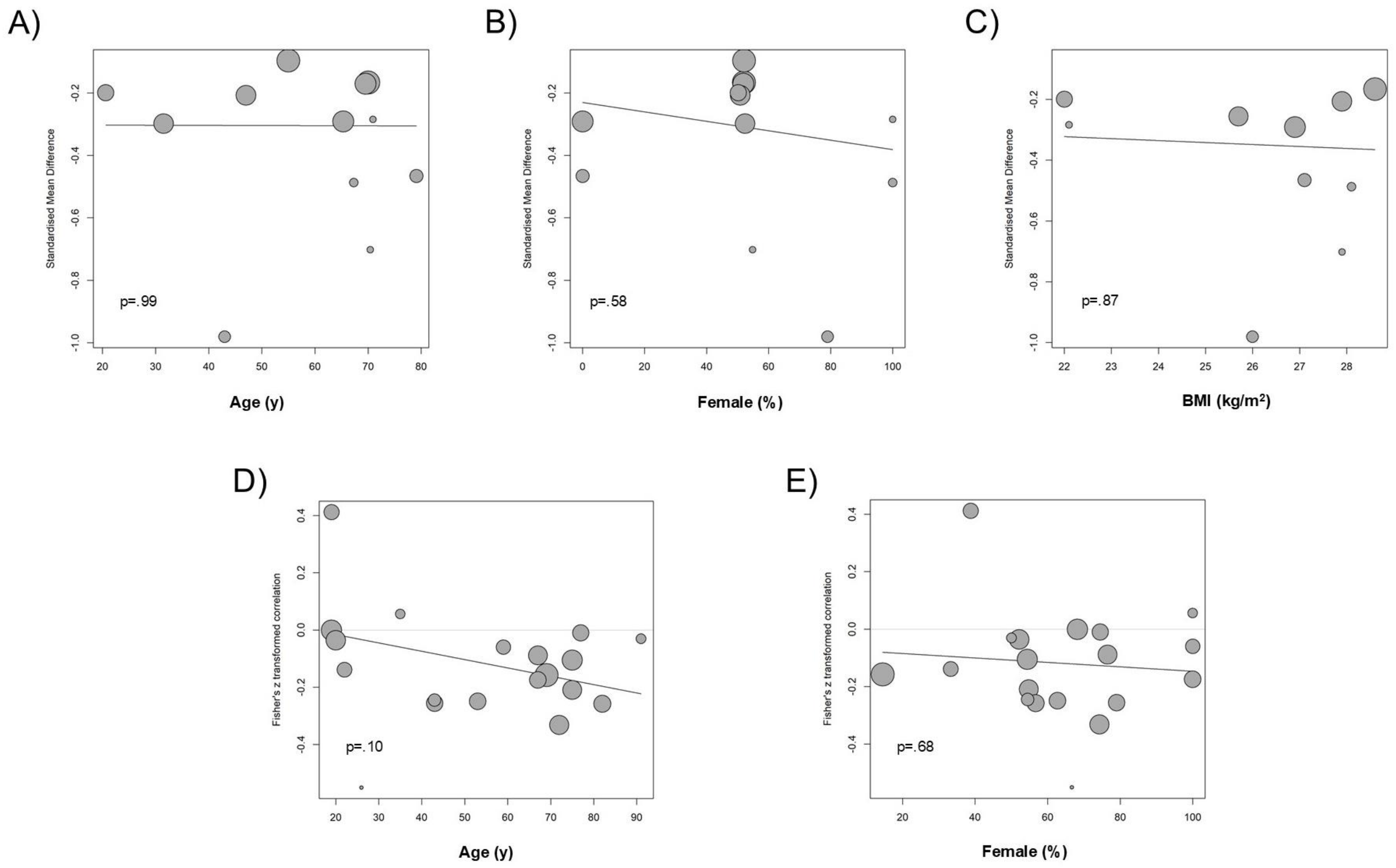

eFigure 1. Meta-regression models on cross-sectional associations between daily steps as a categorical (pooled standardized mean differences: A, B, C) or continuous (pooled correlation coefficients: D, E) variable and depressive symptoms.

© 2024 Bizzozero-Peroni B et al. *JAMA Network Open*.

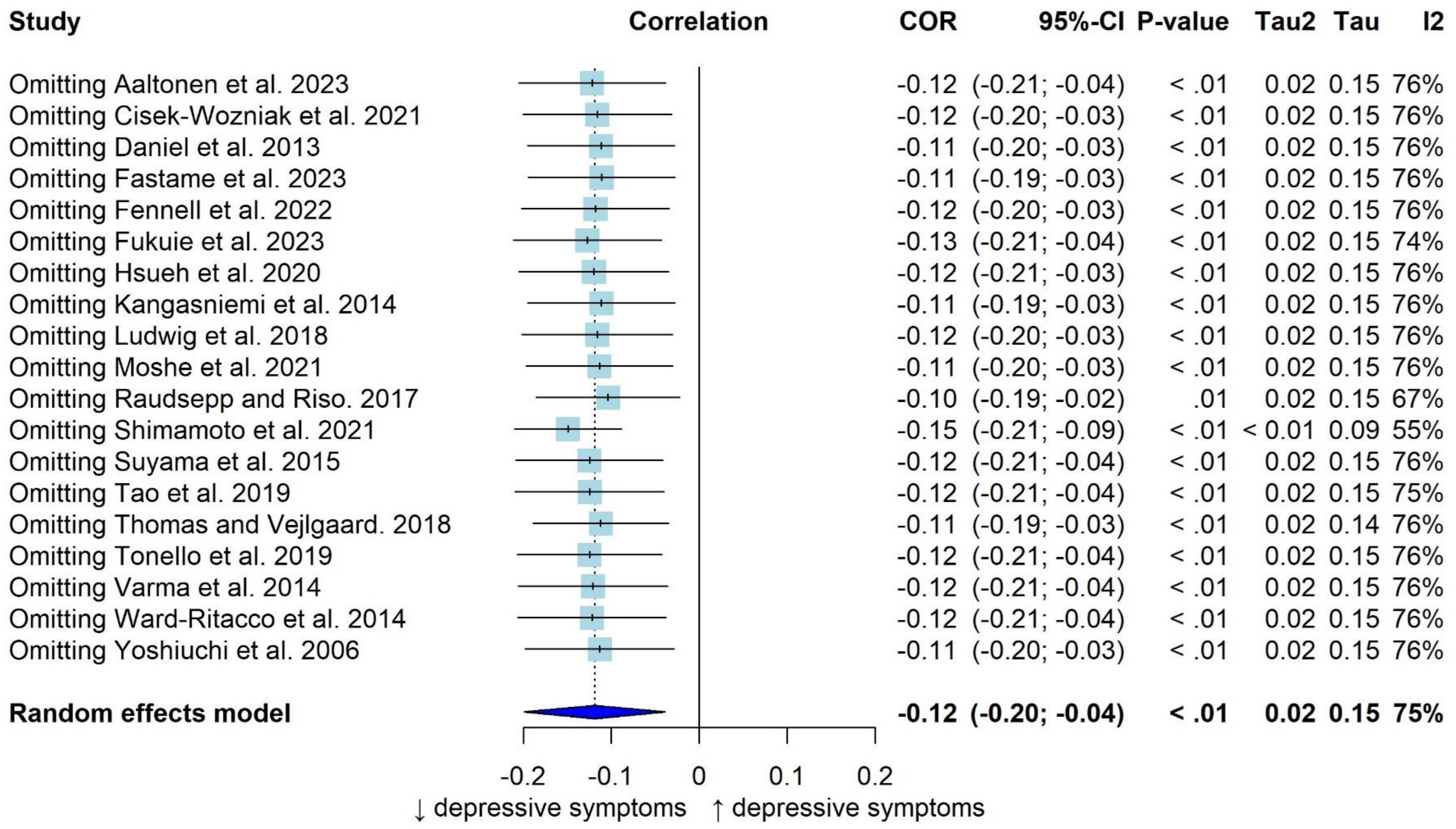

eFigure 2. Sensitivity analysis by removing one-by-one cross-sectional studies of the pooled correlation coefficients.

Abbreviations: CI, confidence interval; CORR, correlation.

© 2024 Bizzozero-Peroni B et al. *JAMA Network Open*.

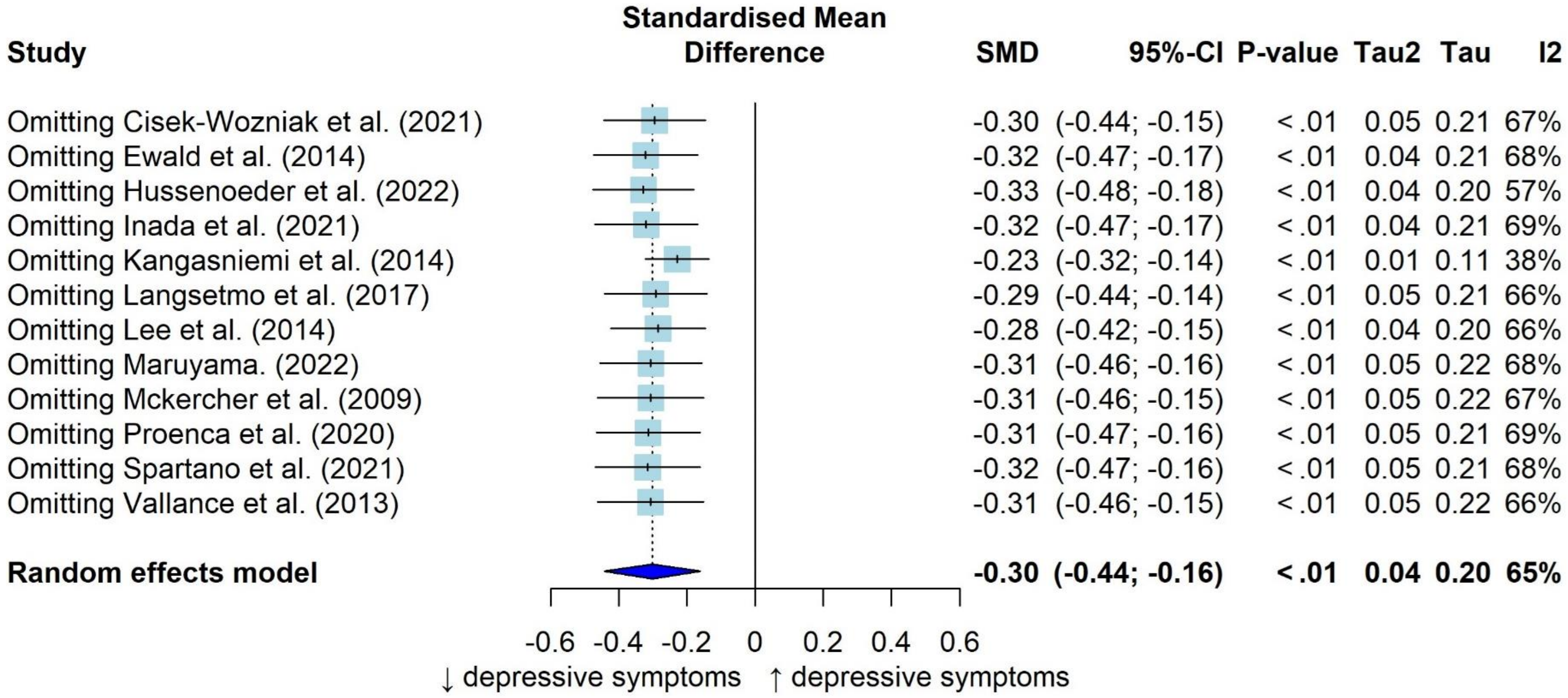

eFigure 3. Sensitivity analysis by removing one-by-one cross-sectional studies from the pooled standardized mean differences of the associations between high (≥7500) *vs*. low (< 7500) levels of daily steps and depressive symptoms.
Abbreviations: CI, confidence interval; SMD, standardized mean difference.

© 2024 Bizzozero-Peroni B et al. *JAMA Network Open*.

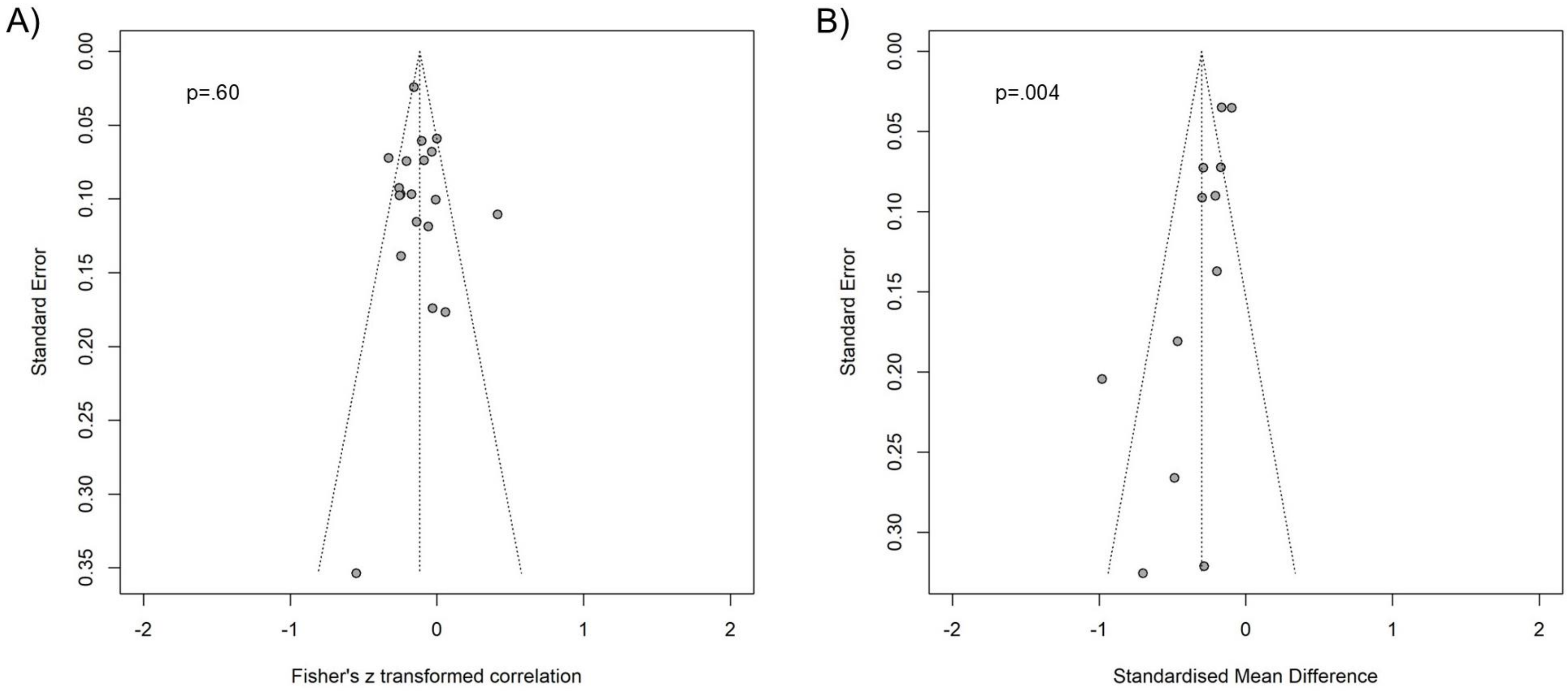

eFigure 4. Publication bias in depressive symptoms from cross-sectional meta-analyses of correlation coefficients (A) and standardized mean differences (B, ≥7500 *vs*. <7500 steps/day).

© 2024 Bizzozero-Peroni B et al. *JAMA Network Open*.
