# Supplementary material for: Daily Step Count and Depression in Adults: A Systematic Review and Meta-Analysis
Source: JAMA Netw Open. 2024 Dec 16;7(12):e2451208. doi: 10.1001/jamanetworkopen.2024.51208 (PMC11650418; doi:10.1001/jamanetworkopen.2024.51208)
Supplement: Supplement 2. — Data Sharing Statement [file jamanetwopen-e2451208-s002.pdf]

Data Sharing Statement

Bizzozero-Peroni. Daily Step Count and Depression in Adults. *JAMA Netw Open*. Published December 16, 2024. doi:10.1001/jamanetworkopen.2024.51208

Data
Data available: No

Additional Information
Explanation for why data not available: This systematic review does not include original data. Data are extracted from the literature and are publicly available.
